# A chemical genetic screen reveals a role for proteostasis in capsule and biofilm formation by *Cryptococcus neoformans*

**DOI:** 10.15698/mic2018.11.656

**Published:** 2018-10-31

**Authors:** François L. Mayer, Eddy Sánchez-León, James W. Kronstad

**Affiliations:** 1 Michael Smith Laboratories, Department of Microbiology and Immunology, University of British Columbia, Vancouver, British Columbia, Canada.

**Keywords:** Cryptococcus neoformans, lithium, proteostasis, capsule, biofilm

## Abstract

Pathogenic microorganisms employ specialized virulence factors to cause disease. Biofilm formation and the production of a polysaccharide capsule are two important virulence factors in *Cryptococcus neoformans, *the fungal pathogen that causes meningoencephalitis. Here, we show that the bipolar disorder drug lithium inhibits formation of both virulence factors by a mechanism involving dysregulation of the ubiquitin/proteasome system. By using a chemical genetics approach and bioinformatic analyses, we describe the cellular landscape affected by lithium treatment. We demonstrate that lithium affects many different pathways in *C. neoformans*, including the cAMP/protein kinase A, inositol biosynthesis, and ubiquitin/proteasome pathways. By analyzing mutants with defects in the ubiquitin/proteasome system, we uncover a role for proteostasis in both capsule and biofilm formation. Moreover, we demonstrate an additive influence of lithium and the proteasome inhibitor bortezomib in inhibiting capsule production, thus establishing a link between lithium activity and the proteasome system. Finally, we show that the lithium-mimetic drug ebselen potently blocks capsule and biofilm formation, and has additive activity with lithium or bortezomib. In summary, our results illuminate the impact of lithium on *C. neoformans,* and link dysregulation of the proteasome to capsule and biofilm inhibition in this important fungal pathogen.

## INTRODUCTION

While relatively common in bacterial pathogens, few fungal pathogens of humans produce an extracellular capsule. An important exception is the opportunistic fungal pathogen, *Cryptococcus neoformans*. Under certain environmental conditions, for example, the elevated temperature of 37°C, alkaline pH, low-iron conditions, reduced nutrient availability, and elevated CO_2 _- levels, *C. neoformans* synthesizes large amounts of polysaccharides that are used to assemble a capsule surrounding the cell body 1,2.

*C. neoformans* is naturally found in the environment and is taken up by humans through inhalation of desiccated yeast cells or spores 3. In most individuals, the fungus is efficiently neutralized by the host immune system. However, in immunocompromised persons, *C. neoformans* can establish a pulmonary infection of the lungs. In a significant number of cases, the fungus can disseminate from the lungs to the brain and cause life-threatening meningoencephalitis 4. It is estimated that 223,100 people become infected with *C. neoformans* each year and that 181,100 patients die from these infections 5. Strikingly, *C. neoformans* is responsible for 15% of all AIDS-associated deaths.

At both common infection sites - the lungs and the brain - *C. neoformans* is primarily present as heavily encapsulated yeast cells 6. In fact, many studies have demonstrated that capsule formation is a major virulence factor in this fungus 7,8,9. The capsule protects cells from oxidative, thermal, enzymatic, and pH stresses, and it protects the fungus from attack by host immune cells 10. Capsule formation is the most important virulence factor in *C. neoformans* but the fungus also produces additional virulence factors including the black pigment melanin, phospholipases, and urease 11. Moreover, *C. neoformans* can form biofilms on medical devices such as catheters 12. Biofilms display enhanced resilience towards antibiotic treatment, and are composed of cell aggregates encased in a matrix of extracellular polysaccharides composed of glucuronoxylomannan (GXM), xylose, mannose, and glucose 13,14. Capsular polysaccharide appears to be required for normal biofilm formation by *C. neoformans*. Pioneering studies by the Casadevall laboratory (Johns Hopkins School of Public Health, USA) have shown that a capsule-deficient *cap59*Δ mutant was unable to form biofilms 15. Complementation of the deletion strain with a *CAP59* wild-type copy restored biofilm formation. The cryptococcal capsule is primarily composed of GXM 16. GXM is also shed by *C. neoformans* during infection and has immune-modulatory properties 17. As mentioned, GXM is a component of the biofilm exopolymeric matrix (EPM), and, therefore, it appears that capsule GXM-shedding may contribute to biofilm formation 12. However, the processes involved in cryptococcal biofilm formation seem to be more complex, because exogenously supplied polysaccharide did not rescue biofilm formation by the acapsular *cap59*Δ mutant 15. Nevertheless, enzyme-linked immunosorbent assay-based spot assays demonstrated that *C. neoformans* biofilms are established via the release of capsular polysaccharide by surface-attached cryptococcal cells 15. Moreover, the binding of shed polysaccharide to solid surfaces was shown to generate a biofilm EPM 15. GXM is a constituent of the EPM, however other sugars not found in capsule GXM, such as ribose and fucose, are also part of the cryptococcal biofilm EPM 18. This suggests that *C. neoformans* biofilms also include polysaccharides other than GXM.

The key role of capsule formation in virulence is illustrated by the fact that *C. neoformans* mutants with absent or defective capsule formation are either avirulent or reduced for virulence in mice 19,20,21. Therefore, understanding the detailed mechanisms of capsule formation in *C. neoformans*, and identifying compounds or drugs that block capsule formation has been the focus of intensive research in the field of cryptococcal pathogenicity. An important pathway that regulates capsule formation in *C. neoformans* is the cyclic adenosine monophosphate-protein kinase A (cAMP-PKA) pathway 22. PKA is composed of the catalytic subunit, Pka1, and the regulatory subunit, Pkr1. Deletion of *PKA1* results in acapsular *C. neoformans* cells, while deletion of *PKR1* leads to hypercapsular cells 23. Using both the *pka1*Δ and *pkr1*Δ mutant, we have previously performed a transcriptomic analysis of these strains grown under capsule-inducing conditions and compared their transcriptomes to a wild-type control 24. These studies identified an array of PKA-regulated factors and pathways potentially contributing to capsule formation including, for example, the heat shock response, vesicle trafficking machinery, and inositol metabolism. As part of further mechanistic studies of inositol metabolism, we used the US Food and Drug Administration (FDA)-approved bipolar disorder drug, lithium. Lithium is known to inhibit inositol metabolism in yeast and, strikingly, exposing *C. neoformans *to lithium chloride (LiCl) potently inhibited capsule formation in a dose-dependent manner 24. However, it is not known whether inositol metabolism really is affected by LiCl treatment in *C. neoformans*, and whether other pathways might also be affected by LiCl and contribute to the anti-capsule activity.

Here, we have analyzed the effects of LiCl on *C. neoformans* by performing a chemical genetics screen. We found that, similar to studies in yeast, lithium has pleiotropic effects in *C. neoformans *and targets multiple pathways and systems, including ion homeostasis, inositol metabolism, the cAMP-PKA pathway, the high osmolarity glycerol (HOG)-pathway, and DNA-repair systems. Strikingly, we discovered that the ubiquitin/proteasome system was also significantly affected by LiCl treatment and represented a central hub of the response to this alkali metal. A combination of genetic and inhibitor studies confirmed a role for proteostasis in virulence factor production by *C. neoformans*. Taken together, our results confirm that proteostasis may represent a novel and attractive antifungal drug target, and that the combination of drugs such as lithium and proteasome inhibitors may be developed further for treatment of infections with this deadly fungus.

## RESULTS

### Lithium inhibits capsule and biofilm formation

**Figure 1:**
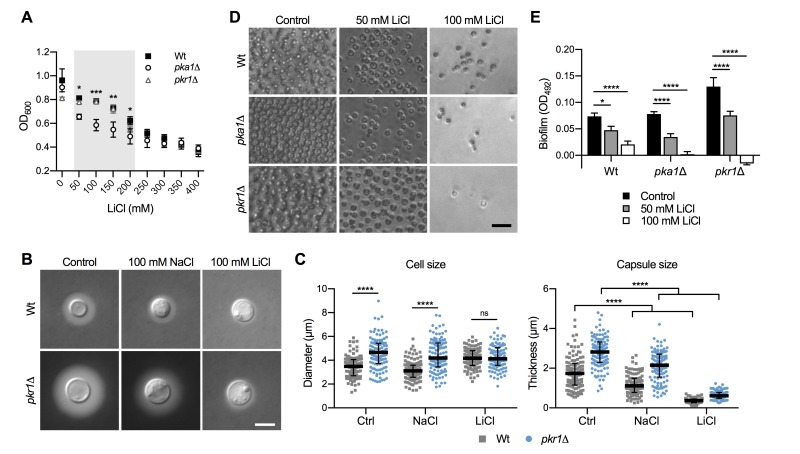
FIGURE 1: Lithium inhibits capsule and biofilm formation. **(A)** Dose-response growth assay for the indicated strains in YPD medium supplemented with increasing concentrations of lithium chloride (LiCl). The strains were incubated for 72 h at 30°C and the optical density was measured at 600 nm (OD_600_). Note that the capsule-deficient *pka1*Δ mutant is significantly more sensitive to LiCl at concentrations ranging from 50 - 200 mM. Wt, wild type (H99S). Results are the mean ± SEM of two independent experiments, each performed in duplicate. *P < 0.05, **P < 0.01, and ***P < 0.001 when comparing *pka1*Δ with the Wt by two-way ANOVA. **(B)** DIC microscopy images of the indicated *C. neoformans* strains grown in CIM (control), CIM + 100 mM NaCl, or CIM + 100 mM lithium chloride for 48 h and stained with India ink to visualize capsule via dye exclusion. Note that lithium strongly inhibits capsule formation in both the H99S Wt and the hypercapsular *pkr1*Δ mutant. Wt, wild type; NaCl, sodium chloride; LiCl, lithium chloride. Scale bar, 5 µm. **(C)** Quantification of cell diameter and capsule thickness for cells from panel B. The experiment was performed twice, and at least 100 cells were analyzed per strain and condition. Grey squares and blue circles indicate individual data points from both experiments, and the black bar indicates the median ± interquartile range. Ctrl, control. ns, not significant. ****P < 0.0001 by two-way ANOVA. **(D)** Brightfield microscopy images of the indicated *C. neoformans* strains grown under biofilm-inducing conditions without (control) or with LiCl for 48 h. Scale bar, 20 µm. **(E)** Quantification of biofilms from panel D by XTT reduction assay. OD_492_, optical density at 492 nm. Results are the mean ± SEM of two independent experiments, each performed in sextuplicate. *P < 0.05, and ****P < 0.0001 by two-way ANOVA.

We previously demonstrated that the *C. neoformans* *pka1*Δ mutant shows increased sensitivity to lithium compared to the wild type on solid YPD medium 24. Our first objective of the current study was to analyze the impact of a range of different lithium chloride (LiCl) concentrations on growth of the *pka1*Δ mutant in liquid YPD medium. We found that concentrations of 50 mM, 100 mM, 150 mM, and 200 mM LiCl resulted in significantly reduced growth of *pka1*Δ compared to the wild-type control (Figure 1A). We also included a *pkr1*Δ mutant in this screen and observed no difference in growth compared to the wild type. These results confirm that Pka1 is required for proper tolerance towards elevated LiCl concentrations. Because the most significant difference in growth between the *pka1*Δ mutant and the wild type was detected at 100 mM LiCl, we chose this concentration for our subsequent experiments on capsule and biofilm formation.

Capsule formation by both the wild-type strain and* pkr1*Δ mutant was strongly inhibited in capsule-induction medium (CIM) supplemented with 100 mM LiCl (Figure 1B). Importantly, supplementation of CIM with 100 mM NaCl only moderately reduced capsule formation by both strains, indicating that LiCl may exert its anti-capsule effect by (a) mechanism(s) distinct from mere induction of ionic stress. As expected, quantification of cell and capsule sizes revealed a larger cell diameter and greater capsule thickness for *pkr1*Δ cells compared to wild-type cells under control conditions (Figure S1 and Figure 1C). Exposure to NaCl did not affect the cell sizes of both strains, but moderately inhibited capsule production (Figure 1C). Confirming our microscopic observations, LiCl strongly suppressed capsule formation by both strains, with *pkr1*Δ cells still forming slightly larger capsules compared to the wild-type strain (Figure 1C). These results indicate that lithium inhibits capsule production involving (a) mechanism(s) distinct from ionic stress.

Because similar polysaccharides are present in both the capsule and in the extracellular matrix of *C. neoformans* biofilms, and because GXM-shedding is essential for biofilm formation, we hypothesized that LiCl may also affect cryptococcal biofilm production. Indeed, we found that LiCl reduced *in vitro* biofilm formation by wild type, *pka1*Δ, and *pkr1*Δ cells in a dose-dependent manner (Figure 1D). We noted that biofilms formed by wild type and *pkr1*Δ cells displayed similar morphologies of clustered cells separated by extracellular matrix. However, cells within biofilms formed by the *pka1*Δ mutant appeared to be smaller in size and spatially closer together (Figure 1D). Quantification by an XTT reduction assay confirmed that LiCl dose-dependently reduced biofilm formation by all three strains (Figure 1E). Compared to the wild-type strain, the *pka1*Δ and *pkr1*Δ mutants were more sensitive to LiCl, and a concentration of 100 mM LiCl completely prevented biofilm formation by these two strains (Figure 1E). In contrast, 100 mM NaCl had no significant effect on biofilm formation by wild type and *pkr1*Δ cells compared to control conditions (Figure S2A and S2B). Biofilm formation by *pka1*Δ was significantly reduced in the presence of NaCl; however, this reduction was not as dramatic as for *pka1*Δ cells exposed to 100 mM LiCl (Figure S2A, S2B and 1E). Together these results show that, besides its anti-capsular activity, LiCl inhibits cryptococcal biofilm formation by (a) mechanism(s) that is independent of mere osmotic or ionic stress induction.

### Phosphoglucomutase does not appear to be a major target of lithium in *C. neoformans*

**Figure 2:**
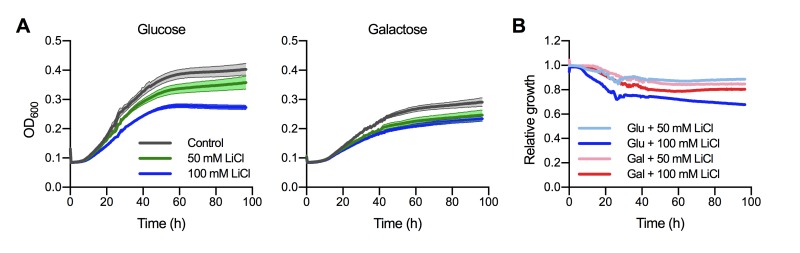
FIGURE 2: Phosphoglucomutase does not appear to be a major target of lithium in *C. neoformans*. **(A)** Growth curve analysis for *C. neoformans* H99S in YNB medium containing either 2% glucose or 2% galactose as sole carbon source at 30°C in the absence (control) or presence of lithium chloride (LiCl). Solid lines represent the means of results from two independent experiments, each performed in quadruplicate, and shaded areas represent the standard errors of the means. **(B)** Relative growth data from panel A. Results were plotted relative to the untreated controls. Note that cells grown in galactose were less sensitive to elevated LiCl concentrations compared to cells grown in glucose. Glu, glucose; Gal, galactose.

Previous studies have demonstrated that lithium potently inhibits phosphoglucomutase in the baker’s yeast *Saccharomyces cerevisiae *25. As a key component of galactose metabolism, phosphoglucomutase catalyzes the reversible isomerization of glucose-1-phosphate to glucose-6-phosphate. *S. cerevisiae* cells grown in YNB medium with 2% galactose as sole carbon source display dramatically enhanced sensitivity towards elevated LiCl concentrations compared to cells grown in medium with 2% glucose 25,26. Similarly, the major human fungal pathogen *Candida albicans* is exquisitely more sensitive to LiCl when grown with 2% galactose as the sole source of carbon compared with glucose 27. We therefore hypothesized that phosphoglucomutase may also be a major target of lithium in *C. neoformans*. To investigate this, we performed growth curve analyses of the *C. neoformans* wild-type strain cultured in YNB medium containing either 2% glucose or 2% galactose and supplemented with different concentrations of LiCl. Under control conditions, fungal cells cultured in glucose grew better than those cultured in galactose (Figure 2A). LiCl inhibited the growth of *C. neoformans* in both media in a dose-dependent manner. However, in contrast to the findings described above for *S. cerevisiae* and *C. albicans*, galactose-grown *C. neoformans* cells, if anything, appeared to be less susceptible to LiCl compared to their glucose-grown counterparts (Figure 2A). We next plotted the relative growth values of these experiments for easier comparison. This approach clearly revealed that galactose-grown *C. neoformans* cells were indeed less susceptible to elevated lithium concentrations compared to cells grown in glucose (Figure 2B). In summary, these results show that in contrast to other fungi, phosphoglucomutase does not appear to be a major target of lithium in *C. neoformans*. Consequently, lithium likely targets alternative factors or pathways in cryptococcal cells.

### A chemical genetics screen identifies genes associated with altered lithium tolerance

**Figure 3:**
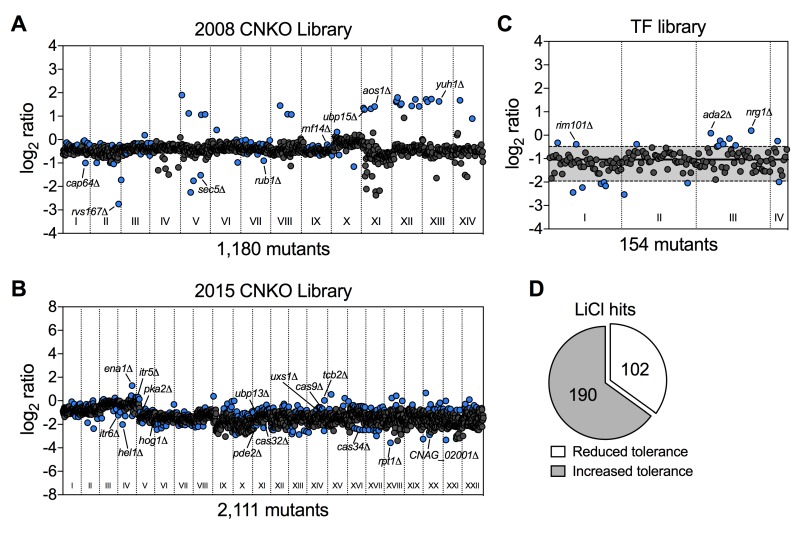
FIGURE 3: Chemical genetic analysis of the impact of lithium on *C. neoformans*. **(A)** Screening results for the 2008 *C. neoformans* knockout (CNKO) library. Results are based on OD_600_ measurements and are plotted as log_2_ ratio of growth in YPD medium supplemented with 100 mM lithium chloride relative to growth in YPD medium only. Roman numerals refer to the 96-well plate numbering as provided by the Fungal Genetics Stock Center (http://www.fgsc.net . Mutants significantly affected by lithium were identified by calculating the mean log_2_-ratio of growth for all mutants of an individual plate and applying a cutoff of ± 1.5-fold the standard deviation (SD). Circles represent individual mutants. Blue colored circles indicate mutants that had significantly reduced (negative log_2_-ratios) or increased (positive log_2_-ratios) tolerance to lithium. Grey circles indicate mutants that did not show altered growth in presence of lithium. **(B)** Screening results for the 2015 CNKO library. See panel A for details. **(C)** Screening results for the transcription factor (TF) mutant library. See panel A for details. Note that for this smaller library, the mean and ± 1.5-fold SD cutoffs were calculated for mutants of all four plates combined instead of for individual plates. The mean is indicated by a solid black line, and the dashed lines indicate the 1.5-fold SD. **(D)** Proportion of mutants identified from the screen with reduced or increased tolerance towards lithium.

Having established that lithium blocks capsule and biofilm formation by *C. neoformans*, and that phosphoglucomutase may not be a major target of lithium in this fungus, we next aimed to obtain a global overview of the cellular effects of LiCl by performing a comprehensive chemical genetics screen. We used three large *C. neoformans* mutant knockout libraries including the 2008 and 2015 CNKO libraries constructed by the laboratory of Dr. Hiten Madhani, and the transcription factor (TF) mutant library constructed by the group of Dr. Yong-Sun Bahn 7,28. In total the three libraries encompassed 3,345 distinct knockout strains representing deletions in approximately half of all predicted open reading frames in *C. neoformans*. We performed our screens in 96-well microtiter plates and cultured each mutant in either YPD (control), or YPD supplemented with 100 mM LiCl for three days at 30°C. We then determined the growth of each mutant in the presence of LiCl relative to the untreated control (Figure S3 and Figure S4). To obtain a comprehensive view of the cellular landscape affected by lithium, we focused on identifying both lithium-hypersensitive and lithium-hypertolerant strains. Furthermore, because of interplate variability, we chose to analyze the results for each 96-well plate individually and applied a cutoff of ± 1.5-fold the standard deviation of the mean of the combined results per plate. The majority of mutants from the 2008 CNKO library were modestly reduced in growth in the presence of LiCl compared to the control condition, indicated by log_2_-ratio values below zero (Figure 3A). Some mutants however were found to be either hypersensitive or hypertolerant to lithium. These strains included, for example, the known capsule-deficient mutant *cap64*Δ, the secretion-defective mutants* rvs167*Δ and *sec5*Δ, and the ubiquitin/proteasome system-associated mutants* rub1*Δ, *ubp15*Δ, *rnf14*Δ,* aos1*Δ and* yuh1*Δ (Figure 3A). Screening the 2015 CNKO library led to the identification of further known and predicted capsule-defective mutants, including *cas9*Δ, *cas32*Δ, *cas34*Δ, and* uxs1*Δ (Figure 3B). Furthermore, mutants with defects in the cAMP/PKA pathway (*pka2*Δ, *pde2*Δ), inositol metabolism (*itr5*Δ, *itr6*Δ and* CNAG_02001*Δ), ion homeostasis (*ena1*Δ and *hog1*Δ), endoplasmic reticulum - plasma membrane tethering (*tcb2*Δ), and the ubiquitin/proteasome system (*hel1*Δ, *ubp13*Δ, and *rpt1*Δ) were identified (Figure 3B). Finally, screening the TF-library led to the identification of further mutants previously published to have impaired capsule production. For example, *rim101*Δ, *ada2*Δ, and *nrg1*Δ were all shown to be hypertolerant to lithium (Figure 3C). Interestingly, when we analyzed the total numbers of hypersensitive and hypertolerant mutants identified from the three libraries, we found that ~1/3 of strains were hypersensitive to lithium (Table S1), while the remaining majority, ~2/3, were hypertolerant (Table S2, Figure 3D). In total, our screen identified 292 mutants with an altered response to lithium.

### Lithium affects multiple pathways

**Figure 4:**
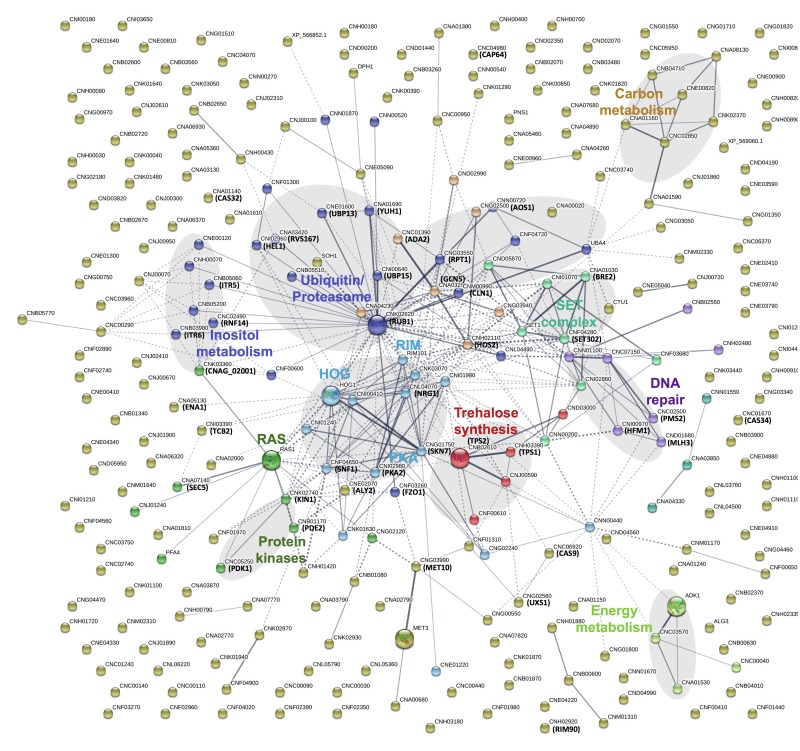
FIGURE 4: A STRING analysis reveals that multiple cellular pathways and activities are regulated by lithium in *C. neoformans*, including the ubiquitin/proteasome system. The corresponding proteins of the 292 mutants found to be hypersensitive or hypertolerant to lithium were subjected to a STRING analysis using default settings. Network edges are based on confidence with line thickness indicating the strength of data support. Network clusters are color-coded and labeled. Note that the network was built based on data from *C. neoformans* strain JEC21. In some cases, the corresponding gene names for *C. neoformans* strain H99 are indicated. HOG, high osmolarity glycerol; PKA, protein kinase A; RAS, rat sarcoma.

In order to obtain a more detailed overview of the pathways and activities affected by lithium, we next conducted a STRING-analysis for the corresponding protein sequences of the 292 deletion mutants identified in our screen. Strikingly, this analysis revealed that most of the pathways known to be affected by lithium in yeast cells were also impacted in *C. neoformans * (Figure 4) 29,30,31. Common regulated functions included the PKA pathway, the HOG pathway, inositol and trehalose metabolism, and DNA repair mechanisms. Furthermore, we identified carbon and energy metabolism, several protein kinases, the SET complex, and the ubiquitin/proteasome system to be affected by lithium in *C. neoformans * (Figure 4). We noticed that the capsule-associated mutants *cap64*Δ, *cas9*Δ, *cas32*Δ, *cas34*Δ, and *uxs1*Δ did not assemble into specific clusters, likely due to their largely unknown cellular functions.

Next, we performed a gene ontology (GO) term analysis for enrichment of molecular functions in our set of lithium-targeted factors. This analysis revealed significant enrichment of ion and anion-specific binding, ion transport, and ion channel activity, indicating that, as expected, lithium perturbs ion homeostasis (Figure S5). Further enriched functions comprised transcription factor activities and RNA-related processes, again fitting with previously described effects of lithium on yeast cells 29,32. Importantly, we also detected a significant, 5-fold enrichment of the GO term category ‘ubiquitinyl hydrolase activity’, confirming that the ubiquitin/proteasome system is a likely target of lithium in *C. neoformans* (Figure S5). Because proteostasis is an emerging theme in cryptococcal capsule regulation, we decided to focus our further analyses on this cellular pathway 33.

### Proteostasis contributes to capsule and biofilm formation by *C. neoformans*

**Figure 5:**
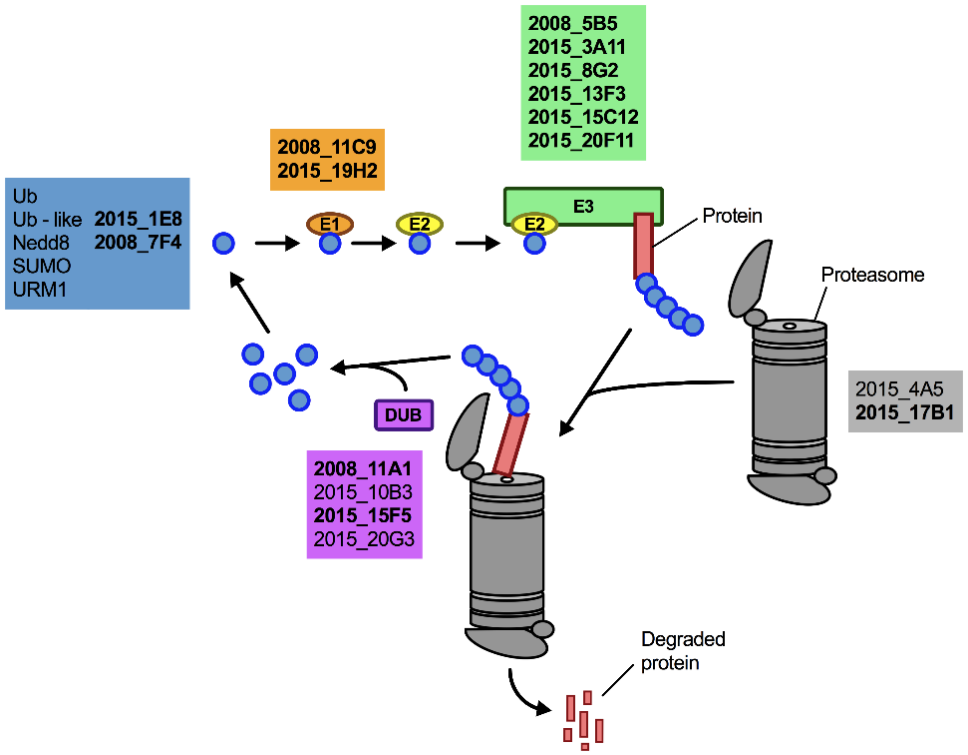
FIGURE 5: The ubiquitin/proteasome-associated mutants identified in the lithium screen have defects in proteins implicated in several stages of the ubiquitin/proteasome system. The strains defective in proteins belonging to a specific functional category of the ubiquitin/proteasome system (for example E1, or DUB) are color-coded and indicated based on their strain designation at the Fungal Genetics Stock Center (http://www.fgsc.net see Table 1). Strains in bold are those that were found to be defective in capsule formation (see Figure 6). Ub, ubiquitin; Nedd8, neural precursor cell expressed, developmentally down-regulated 8; SUMO, small ubiquitin-related modifier; URM1, ubiquitin-related modifier 1; E1, ubiquitin-activating enzyme; E2, ubiquitin-conjugating enzyme; E3, ubiquitin ligase; DUB, deubiquitinating enzyme.

Having demonstrated that lithium inhibits capsule and biofilm formation, and affects the ubiquitin/proteasome system, we next analyzed whether individual mutants associated with this system may have dysregulated capsule and biofilm production. In total, our chemical genetics screen identified 16 ubiquitin/proteasome-associated mutants (Table 1). An *in silico* analysis revealed that the corresponding genes encode proteins that are part of almost all stages of the ubiquitin/proteasome system. Specifically, we identified two ubiquitin-like molecules, two E1-activating enzymes, six E3-ligases, two proteasome-associated proteins, and four deubiquitinating enzymes (Figure 5). Notably, the abundance of three of these proteins (Nedd8, Rpt1, and CNAG_00180) was previously shown to be regulated by PKA (Table 1). We confirmed that the 16 proteasome-associated mutants were affected in their response to elevated lithium levels by performing serial spot dilution assays on solid YPD medium supplemented with 100 mM LiCl. Confirming our results for these strains in liquid media (Table 1), we found that some strains displayed enhanced sensitivity to lithium, while others appeared to be as resistant as the respective wild-type controls (Figure S6).

**TABLE 1:** Ubiquitin/proteasome-associated mutants with altered growth in presence of lithium.

**CNKO - collection^a^**	**Plate**	**Well**	**CNAG - number^b^**	**Function / Predicted function^c^**	**Rel. LiCl - growth^d^**
2008	5	B5	CNAG_02164	hypothetical protein, ubiquitin-ligase domain	2.8
2008	7	F4	**CNAG_02827**	ubiquitin-like protein Nedd8 (Rub1)	0.7
2008	11	A1	CNAG_04493	ubiquitin carboxyl-terminal hydrolase 48 (Ubp15)	3.6
2008	11	C9	CNAG_06342	SUMO activating enzyme (AOS1)	3.9
2015	1	E8	CNAG_04816	hypothetical protein, Ubiquitin domain profile	1.5
2015	3	A11	CNAG_04159	E3 ubiquitin-protein ligase (Hel1)	0.3
2015	4	A5	CNAG_07541	proteasome assembly chaperone 2	1.3
2015	8	G2	CNAG_01746	E3 ubiquitin-protein ligase RNF14	1.2
2015	10	B3	CNAG_02395	ubiquitin carboxyl-terminal hydrolase 9/13 (Ubp13)	1.4
2015	13	F3	CNAG_03777	hypothetical protein, E3 ubiquitin-protein ligase domain	0.4
2015	15	C12	CNAG_03807	E3 ubiquitin-protein ligase CCNP1IP1	0.4
2015	15	F5	CNAG_06983	hypothetical protein, ubiquitin carboxyl-terminal hydrolase	0.4
2015	17	B1	**CNAG_07719**	26S protease regulatory subunit 7 (RPT1)	0.2
2015	19	H2	CNAG_00986	activating enzyme of the ubiquitin-like proteins (Uba4)	1.7
2015	20	F11	CNAG_00171	E3 ubiquitin-protein ligase PEX2	4.1
2015	20	G3	**CNAG_00180**	ubiquitin carboxyl-terminal hydrolase L3 (Yuh1)	3.6

^a^CNKO, *Cryptococcus neoformans* knockout, ^b^Proteins in bold were previously shown to be PKA-regulated 33, ^c^Predicted functions are based on data retrieved from FungiDB, ^d^Relative (rel.) lithium chloride (LiCl) growth is expressed as normalized fold change of mutant growth in YPD medium supplemented with 100 mM LiCl compared to growth in YPD medium alone. A value > 1 indicates that a mutant was more tolerant to LiCl compared to the mean of the remaining mutants of an individual 96-well plate, while a value < 1 indicates hypersensitivity towards LiCl.

**Figure 6:**
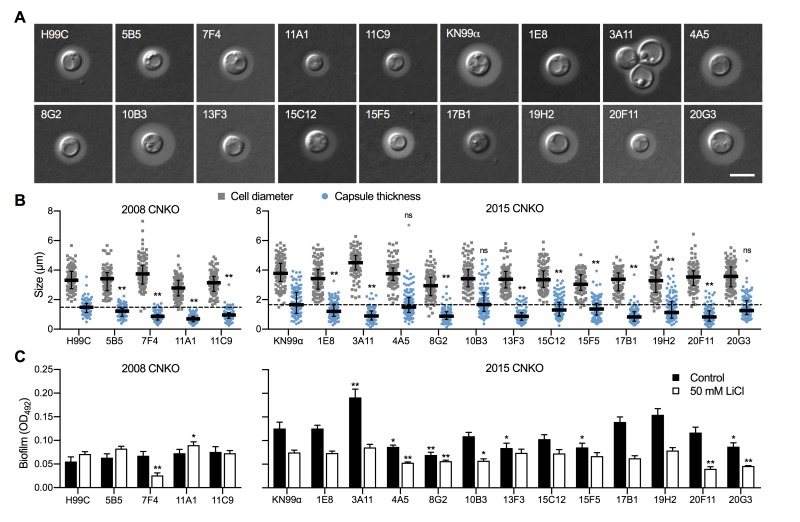
FIGURE 6: The ubiquitin/proteasome system contributes to capsule and biofilm formation. **(A) **DIC microscopy images of the indicated ubiquitin/proteasome-associated *C. neoformans* strains (see Table 1) grown in CIM for 48 h and stained with India ink to visualize capsule via dye exclusion. H99C, Wt control for strains from the 2008 CNKO collection. KN99α, Wt control for strains from the 2015 CNKO collection. Scale bar, 5 µm. **(B)** Quantification of cell diameter (grey squares) and capsule thickness (blue circles) for cells from panel A. The experiment was performed twice, and at least 100 cells were analyzed per strain and condition. The black bar indicates the median ± interquartile range. The dotted black lines indicate the median capsule sizes of the respective Wt controls. ns, not significant. **P < 0.01 by two-way ANOVA compared to the respective Wt. **(C)** Biofilm formation by ubiquitin/proteasome-associated mutants. Indicated strains were grown under biofilm-inducing conditions without (control) or with 50 mM LiCl and quantified by the XTT reduction assay. OD_492_, optical density at 492 nm. Results are the mean ± SEM of three independent experiments, each performed in triplicate. * *P* < 0.05, and **P < 0.01 by *t*-test compared to the respective Wt growth condition.

Next, we analyzed the capacity of each individual mutant to form capsule. Strikingly, we found that all 4 mutants from the 2008 CNKO library, and 9 out of 12 mutants from the 2015 CNKO library had significantly reduced capsule sizes (Figure 6A and 6B). We also performed *in vitro* biofilm assays for the ubiquitin-associated mutants in medium supplemented with or without 50 mM LiCl and found that 2 of the 4 mutants from the 2008 CNKO collection, and 8 out of 12 mutants from the 2015 CNKO collection produced significantly altered biofilms (reduced or enhanced) under either control or lithium-exposed conditions compared to the wild-type controls (Figure 6C). In summary, these results indicate that the ubiquitin/proteasome system contributes to normal capsule and biofilm production in *C. neoformans*.

### The proteasome inhibitor bortezomib potentiates lithium-mediated capsule inhibition

**Figure 7:**
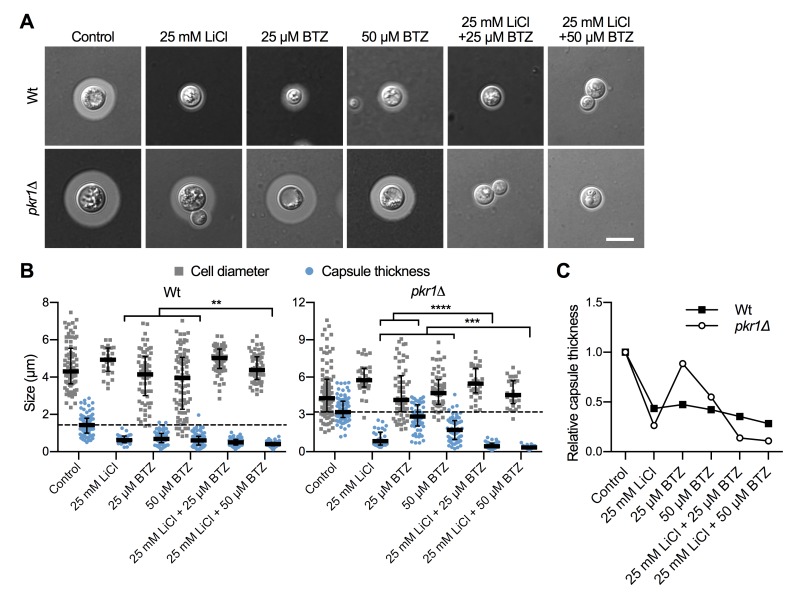
FIGURE 7: Combination of lithium with the proteasome inhibitor bortezomib blocks capsule production. **(A)** DIC microscopy images of the indicated *C. neoformans* strains grown in CIM (control) or CIM supplemented with the indicated concentrations of LiCl and/or bortezomib for 48 h and stained with India ink to visualize capsule. Wt, H99S wild type; BTZ, bortezomib. Scale bar, 5 µm. **(B)** Quantification of cell diameter and capsule thickness for cells from panel A. The experiment was performed twice, and at least 25 cells were analyzed per strain and condition. Small grey squares and blue circles indicate individual data points from both experiments, and the black bar indicates the median ± interquartile range. The dotted black lines indicate the median capsule sizes of the Wt or *pkr1*Δ mutant grown under control conditions. **P < 0.01, ***P < 0.001, and ****P < 0.0001 by *t*-test. **(C)** Relative capsule thickness data from panel B. Results were plotted relative to the untreated controls. Note that capsule formation by the *pkr1*Δ mutant is less affected by BTZ, but more strongly reduced by LiCl compared to the Wt.

The ubiquitin/proteasome system was a prominent cellular component affected by lithium in our screen (Figure 4). We have previously shown that the proteasome inhibitor bortezomib (BTZ) inhibits capsule production by *C. neoformans* in a dose-dependent manner 33. Here, we hypothesized that if lithium indeed targets the cryptococcal proteasome, then using sub-inhibitory concentrations of BTZ together with LiCl should potentially result in strong inhibition of capsule production. Indeed, for the wild-type strain and the hypercapsular mutant *pkr1*Δ, we found that combining 25 mM LiCl with either 25 µM or 50 µM BTZ resulted in significantly reduced capsule sizes compared to treatment of the cells with the individual compounds alone (Figure 7A and Figure 7B). The combination of LiCl and 50 µM BTZ led to a significant reduction of capsule formation in the wild-type strain, while the combination of LiCl with 25 µM or 50 µM BTZ led to significantly reduced capsule sizes in the* pkr1*Δ mutant (Figure 7B). Interestingly, analysis of the data for relative capsule thickness revealed that capsule formation by the *pkr1*Δ mutant was more strongly affected by LiCl treatment, and less affected by BTZ compared to the wild-type strain (Figure 7C). Taken together, these results indicate that LiCl may target the proteasome as part of its activity to reduce capsule production.

### The lithium-mimetic drug ebselen inhibits capsule formation

**Figure 8:**
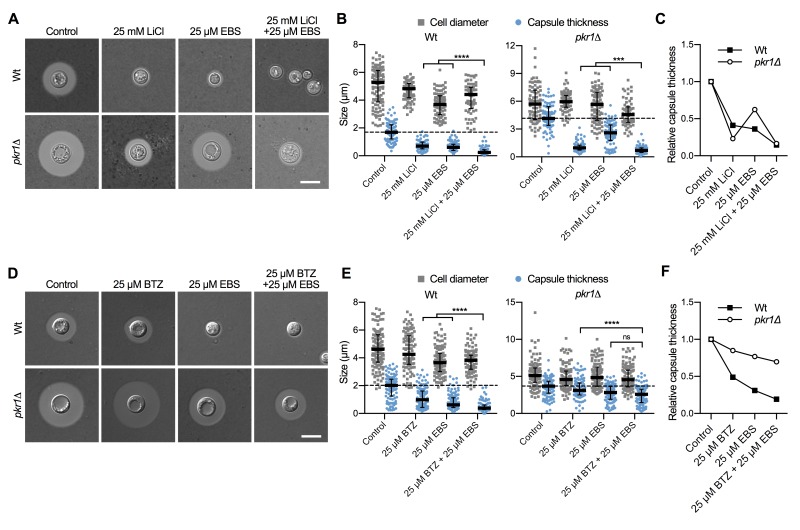
FIGURE 8: The lithium-mimetic drug ebselen inhibits capsule formation, and its activity is potentiated by combination with lithium or bortezomib. **(A)** DIC microscopy images of the *C. neoformans* H99S wild type and the *pkr1*Δ mutant grown in CIM (control) or CIM supplemented with the indicated concentrations of LiCl and/or ebselen for 48 h and stained with India ink to visualize capsule. EBS, ebselen. Scale bar, 5 µm. **(B)** Quantification of cell diameter and capsule thickness for cells from panel A. The experiment was performed twice, and at least 64 cells were analyzed per strain and condition. Grey squares and blue circles indicate individual data points from both experiments, and the black bar indicates the median ± interquartile range. The dotted black lines indicate the median capsule sizes of the Wt or *pkr1*Δ mutant grown under control conditions. ***P < 0.001, and ****P < 0.0001 by *t*-test. **(C)** Relative capsule thickness data from panel B. Results were plotted relative to the untreated controls. Note that capsule formation by the *pkr1*Δ mutant is less affected by EBS but more strongly reduced by LiCl compared to the Wt. **(D)** DIC microscopy images of the *C. neoformans* H99S wild type and the *pkr1*Δ mutant grown in CIM (control) or CIM supplemented with the indicated concentrations of bortezomib and/or ebselen for 48 h and stained with India ink to visualize capsule. BTZ, bortezomib; EBS, ebselen. Scale bar, 5 µm. **(E)** Quantification of cell diameter and capsule thickness for cells from panel D. The experiment was performed twice, and at least 100 cells were analyzed per strain and condition. Small grey squares and blue circles indicate individual data points from both experiments, and the black bar indicates the median ± interquartile range. The dotted black lines indicate the median capsule sizes of the Wt or *pkr1*Δ mutant grown under control conditions. ns, not significant. ****P < 0.0001 by *t*-test. **(F)** Relative capsule thickness data from panel E. Results were plotted relative to the untreated controls. Note that capsule formation by the *pkr1*Δ mutant is less affected by BTZ and EBS compared to the Wt.

Lithium is an important drug for treatment of patients with bipolar disorder. However, at higher concentrations, lithium has considerable toxic side effects. A recent study identified the repurposed drug ebselen (EBS) as a clinically safe lithium mimetic and antioxidant agent 34. Assuming a similar mode-of-action for both lithium and EBS, we analyzed whether a combination of sub-inhibitory concentrations of lithium and EBS would result in potentiated inhibition of capsule formation. Indeed, we found that wild type and* pkr1*Δ cells exposed to both drugs produced significantly reduced capsule sizes compared to exposure to each drug alone (Figure 8A and Figure 8B). Similar to our findings for the combination of LiCl and BTZ, analysis of the relative capsule thickness revealed that capsule formation by* pkr1*Δ was more susceptible to LiCl and more resistant to EBS compared to the wild-type strain (Figure 8C). In summary, these findings demonstrate that EBS has strong potency in inhibiting capsule biosynthesis. Next, we hypothesized that EBS might also target multiple cellular targets including the proteasome system similar to lithium. We therefore exposed *C. neoformans* wild type or *pkr1*Δ mutant cells to EBS, or BTZ alone, or to a combination of EBS and BTZ. The exposure to both drugs resulted in significantly reduced capsule formation by the wild-type strain compared to the single drug treatments (Figure 8D and Figure 8E). However, the drug combination only significantly reduced capsule formation by the *pkr1*Δ mutant compared to the BTZ-only treatment, and not compared to the EBS-only treatment (Figure 8E). Analysis of the relative capsule thickness revealed that capsule formation by the *pkr1*Δ mutant was less susceptible to both BTZ and EBS, compared to the wild-type strain (Figure 8F). In summary, these results demonstrate that EBS potently inhibits capsule formation in a process that may involve the cAMP/PKA pathway and the ubiquitin/proteasome system.

### Clinically relevant concentrations of lithium and EBS impact cryptococcal capsule and/or biofilm formation *in vitro*

**Figure 9:**
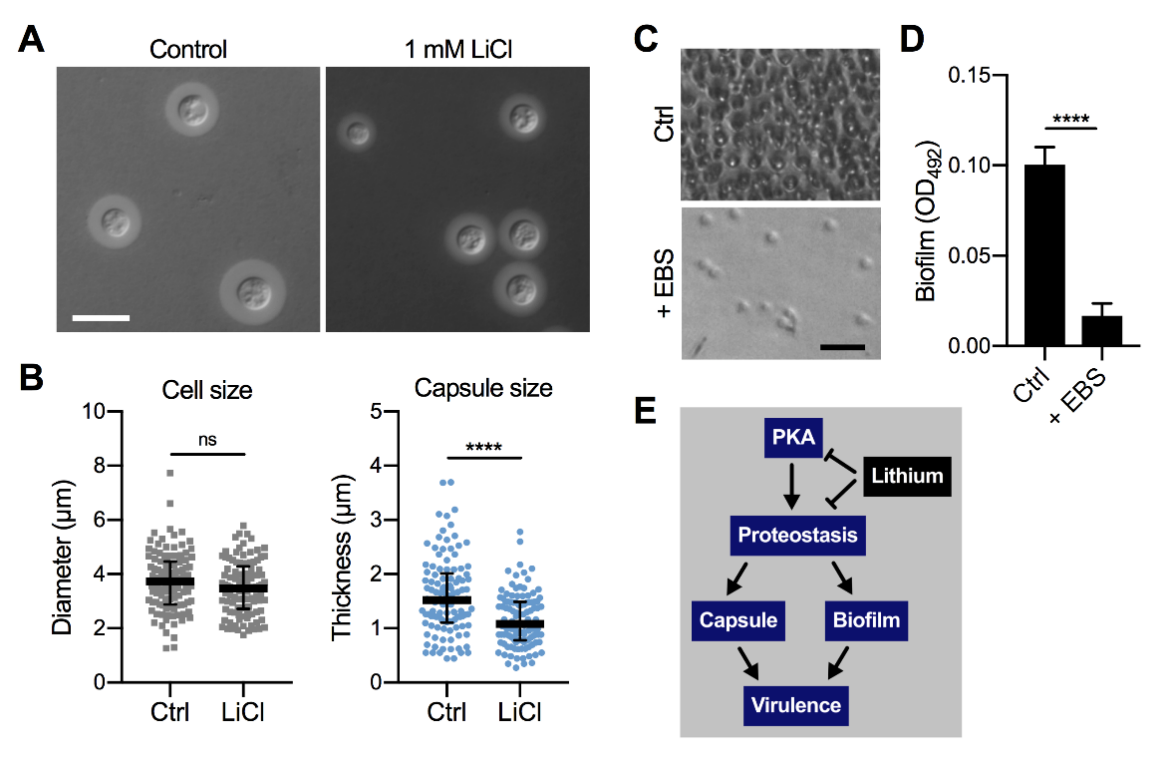
FIGURE 9: Clinically relevant concentrations of lithium chloride and ebselen significantly reduce capsule and/or biofilm formation by *C. neoformans in vitro*. **(A)** DIC microscopy images of *C. neoformans* H99S wild-type cells grown in CIM (control) or CIM supplemented with 1 mM lithium chloride for 48 h and stained with India ink to visualize capsule. Scale bar, 10 µm. **(B)** Quantification of cell diameter and capsule thickness for cells from panel A. The experiment was performed twice, and at least 100 cells were analyzed per strain and condition. Small grey squares and blue circles indicate individual data points from both experiments, and the black bar indicates the median ± interquartile range. ns, not significant. ****P < 0.0001 by *t*-test. **(C)** Brightfield microscopy images of *C. neoformans* H99S grown under biofilm-inducing conditions without (control) or with 20 µg ml^-1^ ebselen (+ EBS) for 48 h. Scale bar, 20 µm. **(D)** Quantification of biofilms from panel C by the XTT reduction assay. OD_492_, optical density at 492 nm. Results are the mean ± SEM of three independent experiments, each performed in triplicate. ****P < 0.0001 by *t*-test. **(E)** Proposed model of proteostasis-dependent capsule and biofilm inhibition by lithium. The PKA-pathway regulates proteasome homeostasis 33, which is required for robust capsule formation and biofilm production, both of which contribute to virulence. Lithium influences both PKA activity and proteostasis, resulting in reduced capsule and biofilm formation.

In order to obtain insight into the global effects of lithium on cellular pathways and activities, we have employed relatively high concentrations of lithium (25 mM to 100 mM LiCl, see experiments described above). However, due to potentially toxic side effects, the final plasma concentrations of lithium used clinically are in the range of only 0.5 mM to 1.2 mM 35,36. We therefore next evaluated the impact of using a clinically relevant dose of 1 mM LiCl on *C. neoformans* capsule and biofilm formation. Interestingly, we found that this concentration of lithium significantly reduced capsule formation by *C. neoformans* wild-type cells compared to untreated control cells (Figure 9A and Figure 9B). Cryptococcal biofilm formation however was not affected by this low dose of LiCl (Figure S7A and Figure S7B).

Although EBS has not yet been approved by the FDA, initial animal trials have used safe EBS doses of 50 - 200 mg kg^-1^ 37,38. We therefore investigated whether a relatively low concentration of 20 µg ml^-1^ EBS would impact *in vitro* biofilm production by *C. neoformans* wild-type cells. Importantly, we found that this dose of EBS strongly inhibited biofilm formation (Figure 9C and Figure 9D). As mentioned above, EBS has been described as a lithium-mimetic drug 34, and we demonstrated that a concentration of 25 µM EBS (~ 6.85 µg ml^-1^) significantly reduced capsule formation by *C. neoformans* and had additive activity with BTZ (Figure 8). Because our results established a role of the ubiquitin/proteasome system in mediating tolerance to LiCl, we next analyzed a potential role of proteostasis in regulating the response to EBS. However, using our set of 16 ubiquitin/proteasome-associated mutants (Table 1), we found that the growth response of these strains differed between exposure to EBS or LiCl (Figure S8). That is, all of the mutants showed reduced growth in the presence of EBS (Figure S8), including those that were more tolerant to LiCl (e.g., CNAG_00171 and CNAG_00180; Table 1). Together, these results demonstrate that clinically relevant concentrations of LiCl or EBS have the capacity to reduce *C. neoformans* capsule and/or biofilm formation *in vitro. *Furthermore, EBS does not appear to evoke the same cellular response compared to lithium with regard to the ubiquitin/proteasome system.

## DISCUSSION

Identifying new targets and novel approaches to target fungal virulence factors are two important aspects of current efforts to discover antifungal drugs 39,40,41,42. These approaches are complemented by strategies to identify new drugs, and repurpose existing drugs as possible antifungals 43,44. Lithium is an FDA-approved drug used to treat persons suffering from bipolar disorder, a brain disorder formerly known as manic depression that causes extreme mood swings. In this study, we have found that lithium potently blocks capsule and biofilm formation by the human fungal pathogen *C. neoformans*, in part via targeting PKA and the ubiquitin/proteasome system (Figure 9E). The ubiquitin/proteasome system is a major mechanism by which eukaryotic proteins are targeted for degradation 45,46. Proteins that are destined for degradation are tagged with ubiquitin in a sequential process involving E1 ubiquitin-activating enzyme, E2 ubiquitin-conjugating enzyme and E3-ubiquitin ligase. Ubiquitin-tagged proteins are then transferred to the proteasome, a multimeric machine in the form of a barrel, through which proteins are pulled and enzymatically degraded 45.

Our finding of an involvement of proteostasis in capsule formation is in full agreement with results from our previous studies in which we analyzed the PKA-regulated *C. neoformans* proteome and identified the ubiquitin/proteasome system as a major component regulating capsule biosynthesis 33. In our present study we independently confirmed these results and showed that exposing cells to lithium and simultaneously targeting proteostasis drastically reduces capsule production. Moreover, mutants with defects in the ubiquitin/proteasome system produced significantly smaller capsules compared to a wild-type control. These results provide further proof for the emerging theme of a critical role of proteostasis in cryptococcal capsule formation. The exact mechanistic underpinnings of proteostasis-mediated capsule regulation however remain to be discovered. For example, it is currently unknown which proteins or pathways may be targeted by the proteasome system during capsule formation.

Hypertonic solutes such as sodium chloride (NaCl) have previously been shown to inhibit capsule formation by *C. neoformans*. Using a high concentration of 1 M NaCl, Jacobson *et al.* demonstrated strong inhibition of capsule due to osmotic stress 47. To investigate whether osmotic stress may also contribute to capsule inhibition by lithium, we studied the effects of a NaCl concentration of 100 mM that matched the treatments with LiCl. Our results indicated that although NaCl indeed reduced capsule formation, this reduction was moderate compared to the effect of lithium. Moreover, 100 mM NaCl had no effect on biofilm formation by wild-type cells. However, we detected an impact of lithium on osmotic stress-related factors such as Hog1, Ena1, and Rim101 in our chemical genetics screen. Taken together these results indicate that although lithium appears to affect ionic homeostasis in *C. neoformans*, osmotic stress does not appear to be the major reason for the observed inhibitory activities on capsule and biofilm formation.

Despite being widely used to treat bipolar disorder, the precise molecular function(s) exerted by lithium are still largely unknown. However, some factors and pathways have been identified as lithium targets in yeast cells, including the enzyme phosphoglucomutase which catalyzes the reversible conversion of glucose-1-phosphate and glucose-6-phosphate and is required for galactose metabolism 25,26. Furthermore, lithium has been shown to block the yeast-to-hyphal transition in *C. albicans* via a mechanism involving inhibition of phosphoglucomutase 27. Therefore, we initially hypothesized that lithium may also target this enzyme in *C. neoformans*. However, our analysis using galactose-grown cells exposed to lithium demonstrated that phosphoglucomutase does not appear to be a major target of lithium in *C. neoformans*. These results point to fundamental differences in the cellular response to this alkali metal between *C. neoformans* and both *C. albicans* and *S. cerevisiae*. The results may also reflect differences in galactose metabolism between the species.

In order to illuminate general effects of lithium on *C. neoformans*, we initially used a relatively high concentration of 100 mM LiCl. Importantly, our growth assays revealed that this LiCl concentration reduced overall growth by approximately 20-30% compared to control conditions without lithium. However, the same lithium concentration reduced capsule and biofilm formation by approximately 70-80%. Therefore, our observation of lithium-mediated virulence factor suppression cannot be explained by simple growth inhibition and instead points to lithium-specific effects.

We used a chemical genetics approach to obtain a more global view of the pathways and factors affected by lithium, and identified several functions including the cAMP/PKA pathway, inositol and trehalose metabolism, DNA repair systems, protein kinases, and the HOG pathway. Importantly, all of these pathways and functions have been previously described as lithium targets 29,30. Surprisingly however, we discovered that the ubiquitin/proteasome system represented a central pathway affected by lithium in *C. neoformans*. This result is quite interesting given that an impact of lithium on the proteasome has previously been described in a murine leukemia cell line 48.

We have found that lithium inhibited both capsule and biofilm formation. Interestingly, several polysaccharides and sugars that constitute the capsule are also found as part of the extracellular biofilm matrix 13,14. We therefore hypothesize that lithium may target a common cellular process, such as possibly proteostasis, that is required for proper assembly of both structures. Indeed, we demonstrated that ubiquitin/proteasome-associated mutants were dysregulated in their ability to form both normal capsules and biofilms. Moreover, to the best of our knowledge, the ubiquitin/proteasome system has not been previously associated with biofilm formation and we hypothesize that proteostasis may contribute to normal biofilm production by *C. neoformans*. In support of this hypothesis, a recent study examining the proteome of *C. neoformans* biofilms identified proteins associated with proteolysis to be specifically upregulated in biofilm cells compared to planktonic cells 49.

The PKA-pathway is known to regulate capsule formation 22,23, and we have previously analyzed the PKA-regulated transcriptome of *C. neoformans*. Lithium was used to block inositol metabolism and was found to inhibit capsule formation in a dose-dependent manner 24. However, whether inositol metabolism was the only pathway targeted by lithium was unknown. In the current study, we have confirmed that lithium inhibits capsule formation by *C. neoformans *and that mutants defective in components of the PKA pathway have altered susceptibility. We also obtained evidence that lithium indeed impacts inositol metabolism, among many other cellular targets. Importantly, we discovered that lithium also dysregulates the ubiquitin/proteasome system. Using a combination of both lithium and the proteasome-inhibiting drug, bortezomib, or the lithium-mimetic drug, ebselen, we demonstrated additive inhibition of capsule formation. Moreover, we established that lithium also inhibits biofilm formation and that mutants in the PKA pathway show differential inhibition. Together, these results link the PKA pathway and proteostasis to the virulence factor-inhibitory activity of lithium.

We noticed that biofilms formed by* pka1*Δ mutant cells displayed a different morphology compared to wild-type biofilms. Biofilm cells of the *pka1*Δ mutant were smaller in size and were in closer spatial proximity to each other. We hypothesize that a defect in polysaccharide secretion required for assembly of the extracellular matrix in the *pka1*Δ mutant may be responsible for this observation.

We identified an involvement of the PKA pathway in regulating the response to BTZ or LiCl, or a combination of both. Since it is known that lithium targets PKA as one of its functions, and that PKA regulates proteostasis 33, these results fit with our proposed model of lithium effects (Figure 9E).

EBS has recently been identified as a safe, lithium-mimetic drug, and it is currently in clinical trials for different (non-fungal) disorders 34. Because EBS showed additive activity with BTZ and LiCl, it may have similar cellular targets as lithium. However, these targets appear to be independent of the ubiquitin/proteasome system, because our mutants with defects in components of the proteasome system displayed divergent growth profiles when comparing growth in the presence of LiCl or EBS. EBS has previously been demonstrated to inhibit cryptococcal growth at relatively low concentrations and may therefore represent an attractive lead compound for development of a novel antifungal agent 50.

We demonstrated that a clinically relevant, low concentration of LiCl significantly reduced *C. neoformans* capsule formation *in vitro*. This result raises the possibility that this low dose of lithium could have clinical relevance for treatment of cryptococcal infections, especially when combined with additional drug therapy. However, *in vivo* lithium efficacy would need to be verified first. The low dose of 1 mM LiCl was not effective in preventing biofilm formation, which is likely due to the intrinsically enhanced resistance of biofilm cells to antimicrobial compounds.

In summary, results of this study link the capsule-inhibiting activity of LiCl to the ubiquitin/proteasome system and suggest that a combination of LiCl and BTZ or EBS may be effective in treating cryptococcosis in the clinic.

## MATERIALS AND METHODS

### Strains and growth conditions

*C. neoformans *variety* grubii *strain H99S (serotype A), strain H99C, and strain KN99α were used as wild-type controls. Other strains used in this study are listed in Table S3. Fungal strains were routinely maintained on YPD agar [1% yeast extract, 2% bacto-peptone, 2% D-glucose, 2% agar]. Overnight cultures were grown in liquid YPD medium in a shaking incubator at 30°C and 180 rpm.

To study the effect of different carbon sources on growth, we used YNB minimal medium [0.17% yeast nitrogen base, 0.5% ammonium sulfate] supplemented with either 2% glucose (YNB-Glu) or 2% galactose (YNB-Gal). A microplate reader (Infinite M200, Tecan) was used for automated growth curve analyses. Overnight fungal cultures were washed twice in sterile phosphate-buffered saline (PBS), and 2 x 10^4^ cells were exposed to 0, 50 or 100 mM LiCl in a final volume of 200 µl YNB-Glu or YNB-Gal in 96-well microtiter plates. Plates sealed with sterile adhesive foil to prevent the evaporation of medium. Growth of the strains was then recorded by measuring the OD_600_ in 30 min intervals for up to 96 hours. Unless noted otherwise, all chemicals were obtained from Sigma-Aldrich.

### Lithium dose-response growth assay

Overnight fungal cultures were diluted 1:40 into YPD medium supplemented with 0, 50, 100, 150, 200, 250, 300, 350, or 400 mM LiCl, and 200 µl were pipetted into wells of a 96-well microtiter plate. The plate was covered with a sterile adhesive foil and incubated at 30°C for 72 h. The OD_600_ was then measured using a microplate reader (Infinite M200, Tecan).

### Serial spot dilution assay

Overnight fungal cultures were washed twice in PBS and cell numbers adjusted to 2 x 10^7^ cells ml^-1^. Next, 10-fold serial dilutions were prepared and five microliters (covering a range of 10^5^ - 10^0^ cells) were spotted onto YPD agar (control) or YPD agar supplemented with 100 mM LiCl. Plates were then incubated at 30°C or 37°C for three days before being photographed.

### Lithium chloride screening

To obtain insight into the global cellular effects of lithium, we performed a chemical genetic screen using the *C. neoformans* 2008 and 2015 CNKO mutant collections from the Madhani laboratory (distributed via the Fungal Genetics Stock Center, http://www.fgsc.net , and the transcription factor (TF) mutant library constructed by the Bahn laboratory 28. The 2008 and 2015 CNKO libraries consist of 1,180 and 2,111 mutants, respectively. The TF-mutant library consists of 322 mutants with deletions in 155 different TFs (with at least two independent deletion strains per TF). In this study, we screened two independent mutants each for 154 of these TFs (a total of 308 mutants) and results are presented as the mean of both mutants. The knockout mutants from the three libraries were grown overnight in sterile, polystyrene, flat-bottom, 96-well microtiter plates (Greiner) in YPD at 30°C. Cultures were then diluted 1:40 in YPD or YPD supplemented with 100 mM LiCl, and plates were incubated in a plastic bag (to avoid medium evaporation) at 30°C for 72 h. The OD_600_ was then measured for each plate using a microplate reader (Infinite M200, Tecan). To obtain relative growth values (expressed as either percent or log_2_-ratios), we divided the values obtained for growth in YPD medium-only by the values for growth in YPD medium containing LiCl. The 2008 and 2015 CNKO mutants significantly affected by lithium were identified by calculating the mean log_2_-ratio of growth for all mutants of an individual 96-well plate and applying a cutoff of ± 1.5-fold the standard deviation (SD). For the smaller TF-library, the mean and ± 1.5-fold SD cutoffs were calculated for mutants of all four plates combined instead of for individual plates.

### *In silico* analysis

In order to analyze our set of lithium-regulated genes, we used the STRING (Search Tool for Retrieval of Interacting Genes/Proteins) program 51. Because the STRING tool utilizes the *C. neoformans* var. *neoformans* JEC21 database as reference, we first retrieved the JEC21 orthologues for each respective *C. neoformans* H99 gene using BLAST (Basic Local Alignment Search Tool) programs at FungiDB and JGI (Joint Genome Institute, http://genome.jgi.doe.gov . The STRING network was built by applying the default settings, including all active interaction sources and a medium confidence value of 0.4. We then used the network 'k-means'-function to analyze the results for potential clusters.

The gene ontology (GO) analysis for our set of genes identified in the lithium screens was performed using FungiDB (http://fungidb.org . Genes were analyzed for GO enrichment in molecular functions using a cutoff of *P* < 0.05 and then ordered according to fold-enrichment.

### Capsule formation

To investigate capsule formation, stationary phase fungal cultures were washed twice in PBS and 40 µl of cells were added to 3 ml low-iron capsule induction medium [CIM; 5 g L^-1^ glucose, 5 g L^-1^ L-asparagine, 0.4 g L^-1^ K_2_HPO_4_, 0.25 g L^-1 ^CaCl_2 _x 2 H_2_O, 0.08 g L^-1 ^MgSO_4_ x 7 H_2_O, 4.78 g L^-1 ^HEPES, 1.85 g L^-1^ NaHCO_3_; dissolved in chelex 100 resin-treated water, pH 7.4] supplemented with or without 100 mM lithium chloride or 100 mM NaCl. Samples were incubated at 30°C and 180 rpm for 48 h. Fungal cells were then stained with India ink to visualize the polysaccharide capsule by DIC microscopy.

For some capsule formation assays, we employed a 96-well plate-based approach. Overnight fungal cultures were diluted 1:40 into CIM or CIM supplemented with either 25 mM LiCl, 25 µM or 50 µM BTZ, 25 µM EBS, or a combination of those. Next, 200 µl per well were added to a sterile 96-well microtiter plate and plates were incubated at 30°C and 180 rpm for 48 h. Each sample was mixed by pipetting up and down, and sub-samples were stained with India ink. Capsule formation was then analyzed by DIC microscopy.

### Inhibitors

For inhibitor studies, the 26S proteasome inhibitor bortezomib (BTZ, New England BioLabs), and the lithium-mimetic drug ebselen (EBS, Sigma-Aldrich) were used. BTZ and EBS were adjusted to 5 mg ml^-1^ in dimethyl sulfoxide (DMSO), aliquoted to small volumes (to avoid multiple freeze-thaw cycles) and kept at -20°C for long-term storage. In our experiments, final DMSO concentrations were < 0.4%.

### Biofilm formation

Biofilm assays were performed based on previously published protocols 14,52, with minor modifications. Briefly, overnight fungal cultures were washed three times in PBS and resuspended in Dulbecco's modified eagle medium (DMEM, Gibco) at a final concentration of 10^6^ cells ml^-1^. For some experiments, DMEM was supplemented with 1 mM, 50 mM, or 100 mM LiCl, 100 mM NaCl, 20 µg ml^-1^ EBS, or a combination of these chemicals. Next, 200 µl of fungal cells were transferred into individual wells of sterile, polystyrene, flat-bottom, 96-well microtiter plates (Corning). Wells with medium only were included as controls. To minimize evaporation, 200 µl sterile water was added to the wells surrounding the test wells. Plates were incubated at 37°C and 5% CO_2_ for 48 h.

### XTT assay

For biofilm analysis, the 2,3-bis(2-methoxy-4-nitro-5-sulfophenyl)-5-[(phenylamino)carbonyl]-2H-tetrazolium hydroxide (XTT) reduction assay was used according to previously published protocols with minor modifications 14,53. An XTT stock solution was prepared in water (1 mg ml^-1^), filter sterilized through a 0.2 µm pore-size filter, aliquoted, and stored at -20°C until use. Prior to each assay, XTT solution was thawed and mixed with a 0.4 mM menadione solution at a ratio of 20:1. Biofilms were washed twice with 100 µl PBS per well to remove unadhered cells, and 200 µl XTT-menadione solution (158 µl PBS mixed with 40 µl XTT solution and 2 µl menadione solution) were added per well. Plates were incubated in the dark for 3.5 h at 37°C. Next, 100 µl of supernatant were transferred to a fresh 96-well plate and the OD_492_ determined in a microplate reader (Infinite M200, Tecan). Values for medium-only controls were subtracted from final sample readings.

### Microscopy

Biofilm formation was analyzed using an inverted microscope (Olympus CKX41). Differential interference contrast (DIC) microscopy was performed with an Axio Imager 2 microscope (Zeiss), and micrographs were captured with an ORCA-flash4.0 LT camera (Hamamatsu) and analyzed with the ZEN software (Zeiss). Quantification of cell diameters and capsule thickness was performed according to Figure S1 using Fiji 54.

### Statistics

Data were visualized and statistically analyzed using GraphPad Prism version 7.0 (GraphPad Software, USA). Statistical tests were performed by two-way analysis of variance (ANOVA) followed by a Bonferroni correction, or by two-tailed, unpaired Student’s *t-*test as indicated in the figure legends. *P*-values of ≤ 0.05 were considered to be significant.

## SUPPLEMENTAL MATERIAL

Click here for supplemental data file.

All supplemental data for this article are also available online at http://microbialcell.com/researcharticles/a-chemical-genetic-screen-reveals-a-role-for-proteostasis-in-capsule-and-biofilm-formation-by-cryptococcus-neoformans/.
