# Supplementary material for: A chemical genetic screen reveals a role for proteostasis in capsule and biofilm formation by Cryptococcus neoformans
Source: Microb Cell. 2018 Oct 31;5(11):495–510. doi: 10.15698/mic2018.11.656 (PMC6244295; doi:10.15698/mic2018.11.656)
Supplement: Supplementary file 1 [file mic-05-495-s01.pdf]

Supplemental Information

A chemical genetic screen reveals a role for proteostasis in capsule and biofilm formation by *Cryptococcus neoformans*

François L. Mayer, Eddy Sánchez-León, James W. Kronstad[#]

Michael Smith Laboratories, Department of Microbiology and Immunology, University of British Columbia, Vancouver, British Columbia, Canada

[#] Corresponding author:
E-mail: kronstad@msl.ubc.ca

Supplementary Figures

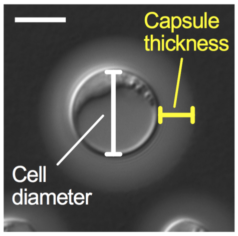

Figure S1: DIC microscopy image of a *C. neoformans* cell grown under capsule inducing conditions and stained with India ink. The cell diameter and capsule thickness were measured as indicated. Scale bar, 5 µm.

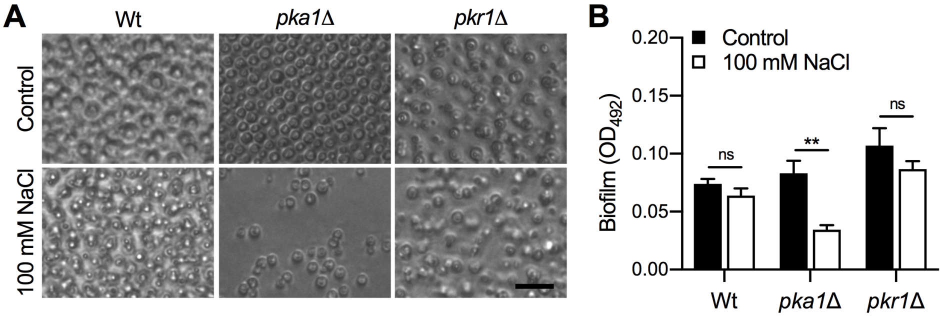

Figure S2: 100 mM NaCl does not affect biofilm formation by Wt (H99S) and *pkr1*Δ strains, but significantly inhibits biofilm formation by the *pka1*Δ mutant. (A) Brightfield microscopy images of the indicated *C. neoformans* strains grown under biofilm-inducing conditions without (control) or with 100 mM NaCl for 48 h. Note that NaCl does not impact Wt and *pkr1*Δ biofilm formation. Wt, wild type. Scale bar, 20 µm. **(B)** Quantification of biofilms from panel A by XTT reduction assay. OD_{492}, optical density at 492 nm. Results are the mean ± SEM of two independent experiments, each performed in sextuplicate. ns, not significant. ** < 0.01 by two-way ANOVA.

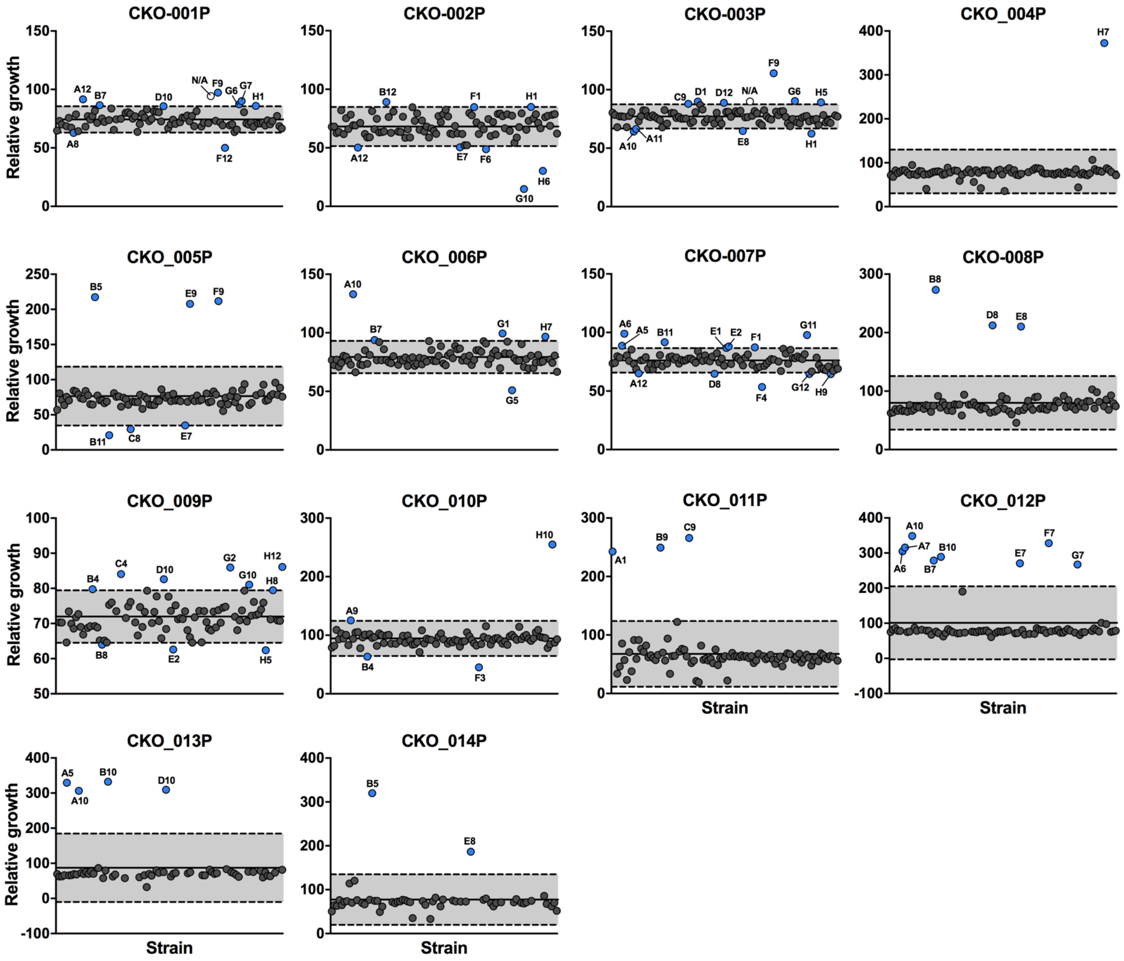

Figure S3: Screening results for the 2008 CNKO library. Results are based on OD_{600} measurements and are plotted as relative growth (YPD medium supplemented with 100 mM lithium chloride versus YPD medium only). Strain IDs refer to mutant designations according to the Fungal Genetics Stock Center (http://www.fgsc.net/). Circles represent individual mutants. Blue colored circles indicate mutants that had significantly reduced or increased tolerance to lithium. Grey circles indicate mutants that did not show altered growth in presence of lithium. White circles indicate mutants with CNAG-numbers that did not match any gene. The mean is indicated by a solid black line, and the dashed lines indicate the 1.5-fold SD.

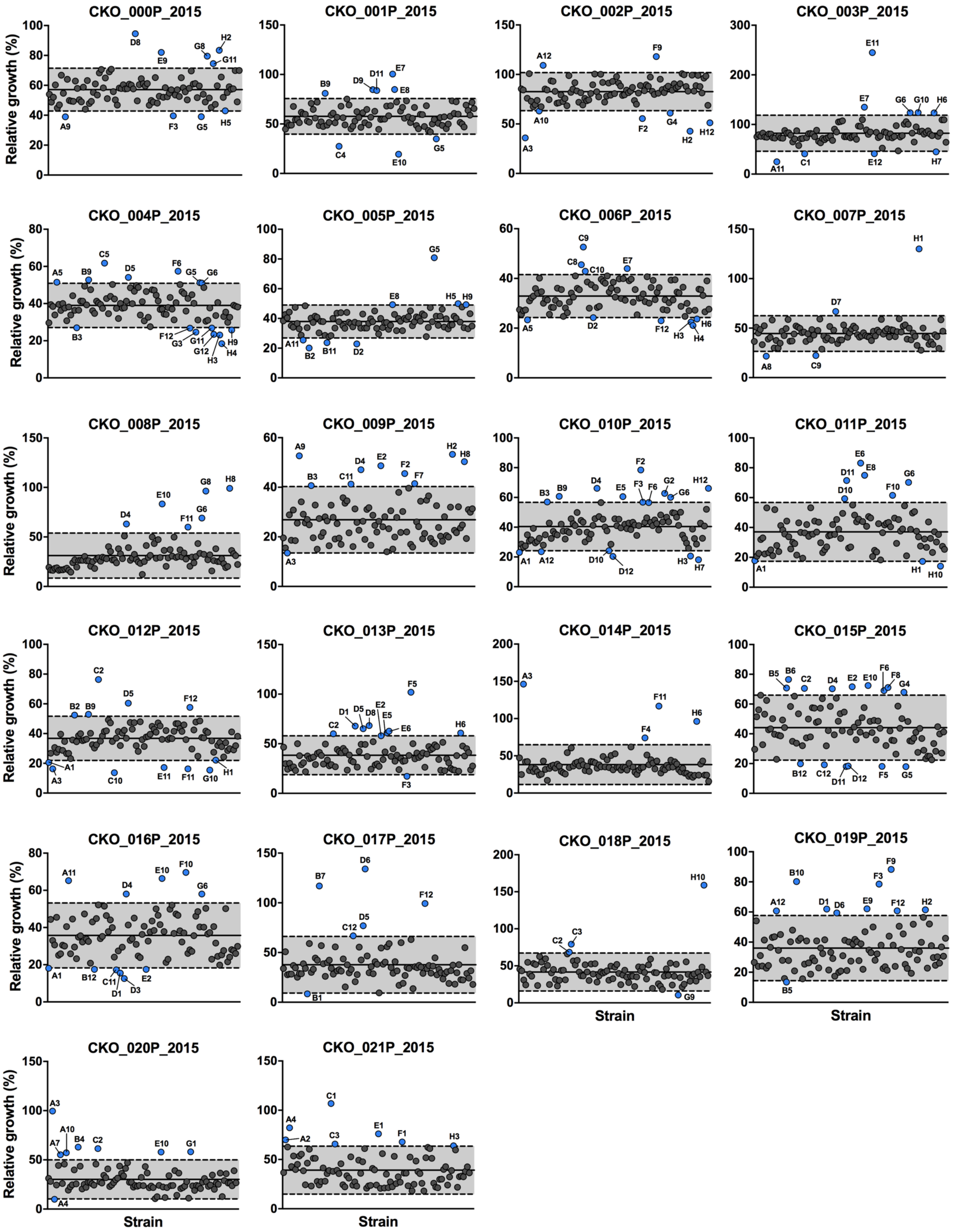

Figure S4: Screening results for the 2015 CNKO library. See Fig. S3 legend for further details.

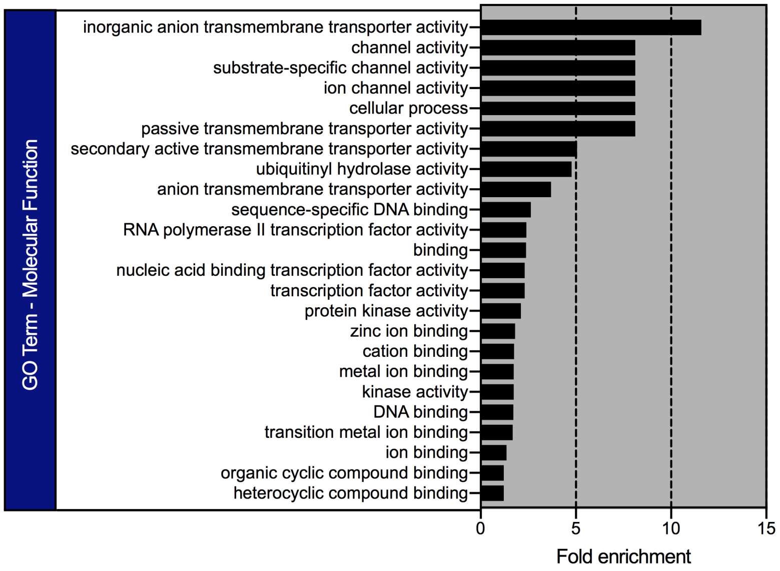

Figure S5: Ion transport activities, ion channel activities, and ubiquitinyl hydrolase activities are significantly enriched in response to lithium treatment. Gene ontology (GO) term analysis of molecular functions significantly enriched in the set of *C. neoformans* mutants found to be affected in their growth by lithium.

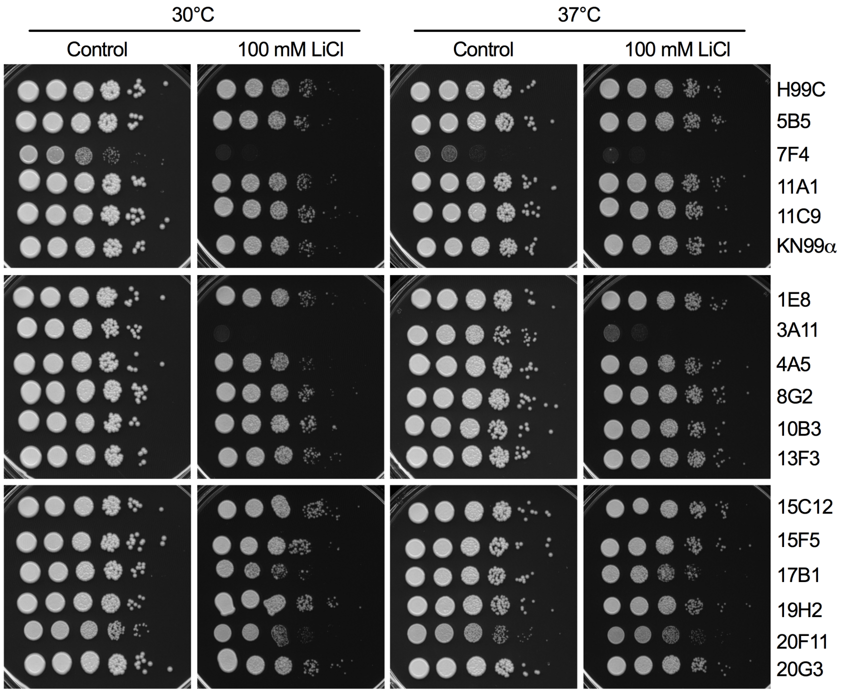

Figure S6: Impact of lithium on growth of the ubiquitin/proteasome-associated mutants. Serial spot dilution assays for the indicated mutants (see Table 1) and respective Wt controls (H99C, and KN99α) on YPD medium supplemented without (control) or with 100 mM LiCl. Plates were incubated at 30°C or 37°C for three days before being photographed.

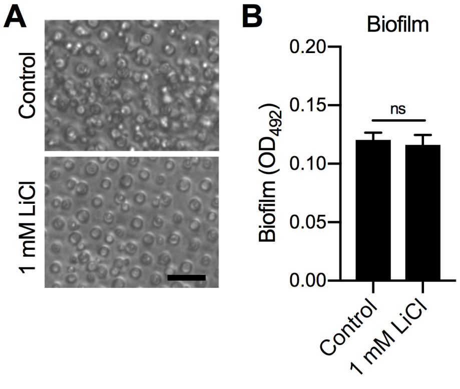

Figure S7: A clinically relevant concentration of lithium does not affect *C. neoformans* biofilm formation. (A) Brightfield microscopy images of *C. neoformans* H99S grown under biofilm-inducing conditions without (control) or with 1 mM lithium chloride for 48 h. Scale bar, 20 µm. **(B)** Quantification of biofilms from panel A by XTT reduction assay. OD_{492}, optical density at 492 nm. Results are the mean ± SEM of three independent experiments, each performed in quadruplicate. ns, not significant by *t*-test.

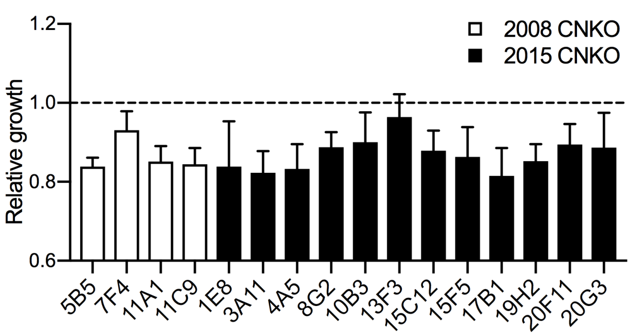

Figure S8: Impact of ebselen on growth of the ubiquitin/proteasome-associated mutants. Results are based on OD_{600} measurements and are plotted as relative growth (YPD medium supplemented with 5 µg ml^{-1} ebselen versus YPD medium only). Strains were incubated at 30°C for three days before being analyzed. White bars indicate mutants from the 2008 CNKO library, and black bars indicate mutants from the 2015 CNKO library. The origin of the *y*-axis was set at 0.6 for better visualization of the data. Note that all strains display reduced growth in presence of ebselen compared to control conditions. Results are the mean ± SD of two independent experiments, each performed in quadruplicate.

Supplementary Tables

Table S1. *C. neoformans* mutants with reduced lithium-tolerance.

Locus	Name	Function
CNAG_00011	N/A	hypothetical protein, has domain(s) with predicted arylformamidase activity and role in tryptophan catabolic process to kynurenine
CNAG_00124	CAS32	hypothetical protein, GDSL-like Lipase/Acylhydrolase family domain
CNAG_00126	N/A	2-deoxy-D-gluconate 3-dehydrogenase
CNAG_00137	N/A	hypothetical protein
CNAG_00150	N/A	peptidase
CNAG_00165	N/A	methylthioadenosine phosphorylase
CNAG_00361	RVS167*	Calmodulin-binding actin-associated protein; roles in endocytic membrane tabulation and constriction, and exocytosis
CNAG_00399	N/A	transformer-2-beta isoform 3, RNA-binding domain
CNAG_00556	CCK1	casein kinase I
CNAG_00736	SEC5*	exocyst protein, exocyst protein, variant
CNAG_00799	N/A	cellulase
CNAG_00805	N/A	hypothetical protein
CNAG_00883	ECM2201	transcription factor
CNAG_01014	N/A	hypothetical protein, Zinc finger, C2H2 type domain
CNAG_01037	N/A	DNA mismatch repair protein MLH3
CNAG_01109	N/A	hypothetical protein
CNAG_01173	PAN1	hypothetical protein, DNA-binding protein
CNAG_01174	N/A	hypothetical protein, Putative threonine/serine exporter
CNAG_01261	N/A	myosin I binding protein, SH3-domain
CNAG_01523	HOG1	mitogen-activated protein kinase
CNAG_01654	CAS34	putative capsule structure designer protein, SGNH hydrolase
CNAG_01713	N/A	hypothetical protein
CNAG_01794	N/A	2-hydroxyacid dehydrogenase
CNAG_01896	N/A	alcohol dehydrogenase (NADP+)
CNAG_01938	KIN1	serine/threonine protein kinase
CNAG_02001	N/A	inositol-polyphosphate 5-phosphatase
CNAG_02047	N/A	hypothetical protein, Acyl-CoA N-acyltransferases domain
CNAG_02115	NHP6B02	nonhistone protein 6
CNAG_02179	N/A	hemolysin
CNAG_02463	N/A	hypothetical protein
CNAG_02480	N/A	cell cycle checkpoint control protein RAD9A
CNAG_02525	N/A	hypothetical protein, Fungal specific transcription factor domain
CNAG_02599	N/A	2,4-dihydroxyhept-2-ene-1,7-dioic acid aldolase
CNAG_02675	N/A	CAMK/CAMKL/GIN4 protein kinase
CNAG_02676	N/A	hypothetical protein, VAM7*, Vacuolar SNARE protein
CNAG_02708	N/A	prenylcysteine oxidase/farnesylcysteine lyase
CNAG_02752	N/A	short-chain dehydrogenase
CNAG_02827	RUB1	ubiquitin-like protein Nedd8
CNAG_02885	CAP64	capsule-associated protein, GDSL-like Lipase/Acylhydrolase family, SGNH hydrolase
CNAG_02915	PDK1	serine threonine protein kinase
CNAG_03060	N/A	multiple drug resistance protein
CNAG_03075	N/A	DNA polymerase delta subunit 3
CNAG_03083	N/A	cupin domain-containing protein
CNAG_03115	N/A	hypothetical protein, Fungal specific transcription factor domain
CNAG_03161	N/A	hypothetical protein
CNAG_03168	MET10*	sulfite reductase (NADPH) flavoprotein alpha-component
CNAG_03409	SKN7	response regulator and transcription factor
CNAG_03413	N/A	alginate lyase
CNAG_03605	N/A	hypothetical protein, Ion transport protein

CNAG_03689	N/A	zf-C3HC4 type zinc finger protein
CNAG_03710	N/A	hypothetical protein, Fungal specific transcription factor domain
CNAG_03764	N/A	integral membrane protein
CNAG_03777	N/A	hypothetical protein, E3 ubiquitin-protein ligase domain
CNAG_03807	N/A	E3 ubiquitin-protein ligase CCNP1IP1
CNAG_03910	ITR6	myo-inositol transporter
CNAG_03911	N/A	hydrolase
CNAG_03981	N/A	palmitoyltransferase PFA4
CNAG_04068	N/A	large subunit ribosomal protein L28e
CNAG_04107	N/A	hypothetical protein
CNAG_04159	HEL1*	E3 ubiquitin-protein ligase (ariadne-1)
CNAG_04215	MET3	sulfate adenylyltransferase
CNAG_04352	ZAP103	zinc-finger protein
CNAG_04433	N/A	CMGC/DYRK/DYRK2 protein kinase
CNAG_04436	N/A	hypothetical protein
CNAG_04461	HFM1	ATP-dependent DNA helicase HFM1/MER3
CNAG_04570	N/A	hypothetical protein, "Winged helix" DNA-binding domain
CNAG_04630	N/A	hypothetical protein, bZIP transcription factor domain
CNAG_04693	AVO1*	target of rapamycin complex 2 subunit
CNAG_04992	N/A	hypothetical protein, SNARE associated Golgi protein domain
CNAG_05093	N/A	hypothetical protein, Homeobox domain
CNAG_05159	N/A	hypothetical protein, hypothetical protein, variant
CNAG_05254	N/A	solute carrier family 35 (UDP-galactose transporter), member B1
CNAG_05292	TPS1	alpha-alpha trehalose-phosphate synthase
CNAG_05337	N/A	alpha-N-arabinofuranosidase, alpha-N-arabinofuranosidase, variant
CNAG_05340	N/A	monosaccharide transporter
CNAG_05372	BLP5	pr4/barwin domain protein
CNAG_05415	N/A	hypothetical protein
CNAG_05444	N/A	NADPH dehydrogenase
CNAG_05678	N/A	membrane protein
CNAG_05737	N/A	oligopeptide transporter 8
CNAG_05842	N/A	cytochrome P450
CNAG_05843	N/A	translation initiation factor 4E
CNAG_05913	N/A	alpha-glucosidase
CNAG_05934	LIV15	translin domain protein
CNAG_06162	N/A	hypothetical protein
CNAG_06232	N/A	transcription factor C subunit 7
CNAG_06324	N/A	zinc finger protein
CNAG_06466	N/A	jumonji domain containing 5
CNAG_06889	N/A	sodium-hydrogen antiporter
CNAG_06983	N/A	hypothetical protein, Ubiquitin carboxyl-terminal hydrolase
CNAG_07313	N/A	hypothetical protein
CNAG_07329	N/A	hypothetical protein, beta-beta-alpha zinc fingers domain
CNAG_07411	RUM1alpha	phd transcription factor
CNAG_07470	PDE2	phosphodiesterase
CNAG_07605	N/A	hypothetical protein
CNAG_07647	N/A	voltage-gated chloride channel protein
CNAG_07662	N/A	hypothetical protein
CNAG_07674	N/A	hypothetical protein, Alpha/beta hydrolase family domain
CNAG_07698	N/A	hypothetical protein
CNAG_07710	N/A	hypothetical protein, NAD(P)-linked oxidoreductase domain
CNAG_07719	RPT1*	26S protease regulatory subunit 7
CNAG_07940	N/A	hypothetical protein, bZIP transcription factor domain

*, originally indicated as N/A in FungiDB, reciprocal BLAST of protein sequence against yeast genome database revealed orthologue

Table S2. *C. neoformans* mutants with increased lithium-tolerance.

Locus	Name	Function
CNAG_00003	N/A	drug transporter
CNAG_00078	N/A	vacuolar protein
CNAG_00125	CRG1	regulator of G-protein signaling
CNAG_00171	N/A	E3 ubiquitin-protein ligase PEX2
CNAG_00173	N/A	hypothetical protein
CNAG_00180	YUH1*	ubiquitin carboxyl-terminal hydrolase L3
CNAG_00192	N/A	hypothetical protein, Zn2/Cys6 DNA-binding domain
CNAG_00289	N/A	hypothetical protein, Acyl-CoA N-acyltransferases domain
CNAG_00291	N/A	hypothetical protein, Rab-GTPase-TBC domain
CNAG_00293	RAS1	Ras-like protein
CNAG_00331	N/A	anon-23da protein, Alpha/beta hydrolase family
CNAG_00375	GCN5	saga complex histone acetyltransferase
CNAG_00390	N/A	myotubularin
CNAG_00397	N/A	2-oxoisovalerate dehydrogenase E1 component, beta subunit
CNAG_00440	SSN801	cyclin subunit of mediator subcomplex
CNAG_00443	N/A	hypothetical protein, Major Facilitator Superfamily domain
CNAG_00452	N/A	isovaleryl-CoA dehydrogenase
CNAG_00492	N/A	hypothetical protein
CNAG_00506	N/A	hypothetical protein
CNAG_00531	ENA1	potassium/sodium efflux P-type ATPase, fungal-type
CNAG_00564	N/A	hypothetical protein, Velvet factor domains
CNAG_00652	N/A	hypothetical protein
CNAG_00658	N/A	hypothetical protein, Ima1 N-terminal domain
CNAG_00714	N/A	hypothetical protein, Thioredoxin-like domain
CNAG_00762	DPH1	diphthamide biosynthesis protein 1
CNAG_00836	N/A	2-hydroxyacid dehydrogenase
CNAG_00986	UBA4	activating enzyme of the ubiquitin-like proteins
CNAG_01079	N/A	2,4-dihydroxyhept-2-ene-1,7-dioic acid aldolase
CNAG_01243	SET101	histone-lysine N-methyltransferase, H3 lysine-4 specific
CNAG_01299	N/A	hypothetical protein, Bestrophin, RFP-TM, chloride channel domain
CNAG_01341	N/A	mannose-6-phosphate isomerase, Cupin domain
CNAG_01387	N/A	hypothetical protein
CNAG_01465	N/A	5' flap endonuclease
CNAG_01489	CAS9	maltose O-acetyltransferase
CNAG_01547	N/A	WD-repeat protein
CNAG_01578	N/A	hypothetical protein, Zinc knuckle domain
CNAG_01580	SCP1	sterol regulatory element-binding protein cleavage-activating protein
CNAG_01611	LIV8	infection related protein of unknown function, Regulator of G protein signaling domain
CNAG_01626	ADA2	transcriptional adapter 2-alpha
CNAG_01746	N/A	E3 ubiquitin-protein ligase RNF14
CNAG_01948	N/A	nuclear protein, Fungal specific transcription factor domain
CNAG_01954	N/A	aldo-keto reductase
CNAG_01970	N/A	hypothetical protein, CCCH zinc finger
CNAG_01971	N/A	hypothetical protein
CNAG_01973	N/A	C2H2 zinc finger protein Zas1A
CNAG_02007	N/A	adenylate kinase 1
CNAG_02009	N/A	extensin
CNAG_02025	N/A	DNA-directed RNA polymerase III subunit RPC3
CNAG_02030	N/A	glyoxal oxidase
CNAG_02044	N/A	hypothetical protein
CNAG_02102	N/A	hypothetical protein
CNAG_02103	N/A	hypothetical protein, hypothetical protein, variant
CNAG_02122	N/A	cytoplasmic tRNA 2-thiolation protein 1

CNAG_02164	N/A	hypothetical protein, ubiquitin-ligase domain
CNAG_02305	N/A	hypothetical protein, Fungal specific transcription factor domain
CNAG_02341	ALY2*	cyclin binding protein
CNAG_02387	N/A	hypothetical protein, Velvet factor domain
CNAG_02395	UBP13*	ubiquitin carboxyl-terminal hydrolase 9/13
CNAG_02427	N/A	hypothetical protein, Isochorismatase-like hydrolase domain
CNAG_02435	BWC2	white-collar transcription factor, blue-light photoresponsive gene
CNAG_02469	N/A	cytoplasmic protein
CNAG_02478	N/A	glycerol dehydrogenase
CNAG_02554	N/A	sugar transporter
CNAG_02658	N/A	hypothetical protein, Cyclin domain
CNAG_02694	N/A	hypothetical protein, hypothetical protein, variant
CNAG_02699	N/A	hypothetical protein
CNAG_02753	LIV13	endoplasmic reticulum protein
CNAG_02776	N/A	hypothetical protein
CNAG_02777	PHO84	phosphate:H symporter, phosphate:H symporter, variant
CNAG_02789	N/A	nitrogen permease regulator 2
CNAG_02864	N/A	hypothetical protein
CNAG_02882	N/A	hypothetical protein
CNAG_02986	YSA1	ADP-ribose pyrophosphatase
CNAG_03044	N/A	hypothetical protein, HD-domain/PDEase-like
CNAG_03080	N/A	fatty acid elongase
CNAG_03114	N/A	hypothetical protein
CNAG_03154	N/A	hypothetical protein, hypothetical protein, variant
CNAG_03173	N/A	DNA damage-binding protein 1
CNAG_03238	N/A	dioxygenase subfamily protein
CNAG_03272	N/A	hypothetical protein, NAD(P)-binding Rossmann-fold domain
CNAG_03322	UXS1	UDP-glucuronic acid decarboxylase
CNAG_03329	N/A	PHD-finger protein
CNAG_03355	TCO4	two-component-like sensor kinase
CNAG_03362	N/A	hypothetical protein
CNAG_03370	N/A	calcium-binding protein NCS-1
CNAG_03401	N/A	hypothetical protein, GATA zinc finger domain
CNAG_03403	N/A	mitochondrial protein
CNAG_03431	N/A	nuclear protein, Fungal specific transcription factor domain
CNAG_03434	N/A	solute carrier family 45, member 1/2/4
CNAG_03452	N/A	AFG1 family mitochondrial ATPase
CNAG_03495	N/A	hypothetical protein, WW domain
CNAG_03517	N/A	NADH dehydrogenase
CNAG_03542	N/A	arginase
CNAG_03632	N/A	hypothetical protein, MAPEG family domain
CNAG_03741	N/A	hypothetical protein, Zn2/Cys6 DNA-binding domain
CNAG_03760	RINT1	hypothetical protein
CNAG_03765	TPS2	trehalose-phosphatase
CNAG_03768	N/A	hypothetical protein, Zn2/Cys6 DNA-binding domain
CNAG_03771	N/A	DNA binding protein Ncp1, DNA binding protein Ncp1, variant
CNAG_03838	N/A	hypothetical protein, MFS general substrate transporter
CNAG_03858	N/A	hypothetical protein
CNAG_03867	N/A	hypothetical protein
CNAG_03902	N/A	transcriptional regulatory protein
CNAG_04024	ITR5	sugar transporter
CNAG_04038	N/A	MFS quinate transporter QutD
CNAG_04098	PDR5-2	ATP-binding cassette, subfamily G (WHITE), member 2, PDR
CNAG_04146	N/A	hypothetical protein, SET domain
CNAG_04162	PKA2	protein kinase A (PKA) catalytic subunit
CNAG_04200	TCB2*	C2 domain-containing protein, Calcium/lipid-binding domain

CNAG_04224	N/A	carboxy-terminal domain RNA polymerase II polypeptide A small phosphatase
CNAG_04432	N/A	hypothetical protein, ARM repeat domain
CNAG_04450	N/A	chromodomain-helicase-DNA-binding protein 1
CNAG_04475	N/A	hypothetical protein, Histidine phosphatase superfamily domain
CNAG_04493	UBP15*	ubiquitin carboxyl-terminal hydrolase 48
CNAG_04514	MPK1	mitogen-activated protein kinase
CNAG_04536	N/A	nicotinamide mononucleotide permease
CNAG_04588	N/A	hypothetical protein, Zn2/Cys6 DNA-binding domain
CNAG_04621	N/A	glycogen(starch) synthase, glycogen(starch) synthase, variant
CNAG_04634	N/A	hypothetical protein
CNAG_04659	N/A	pyruvate decarboxylase
CNAG_04756	N/A	hypothetical protein
CNAG_04759	N/A	hypothetical protein, DENN (AEX-3) domain
CNAG_04760	N/A	cytoplasmic protein
CNAG_04804	SRE1	sterol regulatory element-binding protein
CNAG_04816	N/A	hypothetical protein, Ubiquitin domain profile
CNAG_04836	N/A	nuclear protein, Fungal specific transcription factor domain
CNAG_05038	N/A	hypothetical protein
CNAG_05083	N/A	esterase/lipase
CNAG_05142	N/A	dolichyl-P-Man:Man(5)GlcNAc(2)-PP-dolichyl mannosyltransferase
CNAG_05177	N/A	DNA polymerase kappa subunit
CNAG_05222	NRG1	transcriptional regulator Nrg1
CNAG_05315	N/A	taurine catabolism dioxygenase TauD
CNAG_05343	N/A	hypothetical protein, Arrestin (or S-antigen), C-terminal domain
CNAG_05352	N/A	hypothetical protein, Acetyl-coenzyme A transporter 1 domain
CNAG_05375	N/A	hypothetical protein, Helix-loop-helix DNA-binding domain
CNAG_05401	N/A	hypothetical protein, Armadillo/plakoglobin ARM repeat profile
CNAG_05411	N/A	endoglucanase
CNAG_05422	LIV11	virulence related protein of unknown function
CNAG_05424	N/A	hypothetical protein
CNAG_05431	RIM101	pH-response transcription factor pacC/RIM101
CNAG_05443	N/A	hypothetical protein, Plasma-membrane choline transporter domain
CNAG_05445	N/A	smooth muscle cell associated protein-1 isoform 2
CNAG_05484	N/A	CAMKK/CAMKK-META protein kinase
CNAG_05520	N/A	hypothetical protein
CNAG_05538	JJJ1	hypothetical protein, DnaJ domain protein
CNAG_05563	HOS2	histone deacetylase HOS2
CNAG_05606	N/A	ATP-dependent DNA helicase PIF1
CNAG_05654	RIM90	hypothetical protein, SUR7/PalI family
CNAG_05685	N/A	neutral amino acid transporter
CNAG_05698	N/A	hypothetical protein
CNAG_05742	STP1	putative site-2 protease
CNAG_05781	N/A	dynein light chain LC8-type
CNAG_05835	LIV3	wor1/pac2 family transcription factor
CNAG_05907	N/A	pyruvate carboxylase
CNAG_05929	N/A	MFS transporter, SP family, general alpha glucoside:H+ symporter
CNAG_06092	CLN1	cyclin 1
CNAG_06112	N/A	carbamoyl-phosphate synthase arginine-specific large chain
CNAG_06126	N/A	Pin2-interacting protein X1, G-patch domain
CNAG_06230	N/A	hypothetical protein, Triose-phosphate Transporter family domain
CNAG_06283	LIV4	putative myb-like mRNA polymerase I termination factor
CNAG_06291	FPD1	polysaccharide deacetylase
CNAG_06314	N/A	phosphoribosylamine-glycine ligase
CNAG_06326	N/A	solute carrier family 40 (iron-regulated transporter), Ferroportin1 domain
CNAG_06342	AOS1*	SUMO activating enzyme

CNAG_06343	N/A	hypothetical protein, SOH1 domain
CNAG_06384	N/A	DNA repair protein RAD50
CNAG_06431	N/A	acyl-CoA oxidase
CNAG_06529	N/A	hypothetical protein, Oxidoreductase family, NAD-binding domain
CNAG_06545	N/A	pim1 protein RNA transporter 2
CNAG_06552	SNF1*	CAMK/CAMKL/AMPK protein kinase
CNAG_06561	N/A	allantoate transporter
CNAG_06591	SET302	hypothetical protein, SET domain
CNAG_06617	N/A	hypothetical protein, Calcineurin-like phosphoesterase domain
CNAG_06648	RTF1	mRNA polymerase-associated protein, Plus-3 domain
CNAG_06649	N/A	haloacid dehalogenase, type II
CNAG_06687	N/A	hypothetical protein, Pericentrin-AKAP-450 domain of centrosomal targeting protein
CNAG_06688	FZO1*	mitofusin
CNAG_06739	N/A	dethiobiotin synthase
CNAG_06742	N/A	pre-60S factor REI1, Zinc-finger double-stranded RNA-binding
CNAG_06752	N/A	hypothetical protein, Arsenical pump membrane protein
CNAG_06814	SXI1alpa	hypothetical protein, Homeobox KN domain
CNAG_06925	N/A	arsenical-resistance protein
CNAG_06984	N/A	hypothetical protein
CNAG_07311	BRE2	compass component bre2
CNAG_07368	N/A	calcium activated cation channel protein
CNAG_07524	N/A	AMP deaminase
CNAG_07541	N/A	proteasome assembly chaperone 2
CNAG_07556	N/A	hypothetical protein
CNAG_07599	N/A	DNA mismatch repair protein PMS2
CNAG_07887	N/A	hypothetical protein, WW domain

*, originally indicated as N/A in FungiDB, reciprocal BLAST of protein sequence against yeast genome database revealed orthologue

Table S3. Strains used in this study.

Strain	Genotype	Reference
H99S	Serotype A, *MAT*α	Joseph Heitman, USA [55]
H99C	Serotype A, *MAT*α	Jennifer Lodge, USA [56]
KN99α	wild type	Joseph Heitman, USA [55]
pka1Δ	*pka1::ade2*	Joseph Heitman, USA [23]
pkr1Δ	*pkr1::ura5*	Joseph Heitman, USA [23]

References

23. D'Souza CA, Alspaugh JA, Yue C, Harashima T, Cox GM, Perfect JR, Heitman J (**2001**). Cyclic AMP-dependent protein kinase controls virulence of the fungal pathogen *Cryptococcus neoformans*. **Mol Cell Biol** 21(9): 3179-3191. doi: 10.1128/MCB.21.9.3179-3191.2001.

55. Janbon G, Ormerod KL, Paulet D, Byrnes EJ, 3rd, Yadav V, Chatterjee G, Mullapudi N, Hon CC, Billmyre RB, Brunel F, Bahn YS, Chen W, Chen Y, Chow EW, Coppee JY, Floyd-Averette A, Gaillardin C, Gerik KJ, Goldberg J, Gonzalez-Hilarion S, Gujja S, Hamlin JL, Hsueh YP, Ianiri G, Jones S, Kodira CD, Kozubowski L, Lam W, Marra M, Mesner LD, et al. (**2014**). Analysis of the genome and transcriptome of *Cryptococcus neoformans* var. *grubii* reveals complex RNA

expression and microevolution leading to virulence attenuation. **PLoS Genet** 10(4): e1004261. doi: 10.1371/journal.pgen.1004261.

56. Nelson RT, Hua J, Pryor B, Lodge JK (**2001**). Identification of virulence mutants of the fungal pathogen *Cryptococcus neoformans* using signature-tagged mutagenesis. **Genetics** 157(3): 935-947. PMID: 11238384.
